# Different seminal ejaculated fractions in artificial insemination condition the protein cargo of oviductal and uterine extracellular vesicles in pig

**DOI:** 10.3389/fcell.2023.1231755

**Published:** 2023-10-06

**Authors:** S. M. Toledo-Guardiola, C. Luongo, L. Abril-Parreño, C. Soriano-Úbeda, C. Matás

**Affiliations:** ^1^ Departamento de Fisiología, Facultad de Veterinaria, Campus de Excelencia Mare Nostrum Universidad de Murcia, Murcia, Spain; ^2^ Departamento de Medicina, Cirugía y Anatomía Veterinaria, Universidad de Léon, León, Spain; ^3^ Instituto Murciano de Investigación Biosanitaria Pascual Parrilla (IMIB-Arrixaca), Murcia, Spain

**Keywords:** ejaculate fractions, extracellular vesicles, insemination, oviductal fluid, pregnant sows, uterine fluid

## Abstract

The seminal plasma (SP) is the liquid component of semen that facilitates sperm transport through the female genital tract. SP modulates the activity of the ovary, oviductal environment and uterine function during the periovulatory and early pregnancy period. Extracellular vesicles (EVs) secreted in the oviduct (oEVs) and uterus (uEVs) have been shown to influence the expression of endometrial genes that regulate fertilization and early embryo development. In some species, semen is composed of well-separated fractions that vary in concentration of spermatozoa and SP composition and volume. This study aimed to investigate the impact of different accumulative fractions of the porcine ejaculate (F1, composed of the sperm-rich fraction, SRF; F2, composed of F1 plus the intermediate fraction; F3, composed of F2 plus the post-SRF) on oEVs and uEVs protein cargo. Six days after the onset of estrus, we determined the oEVs and uEVs size and protein concentration in pregnant sows by artificial insemination (AI-sows) and in non-inseminated sows as control (C-sows). We also identified the main proteins in oEVs and uEVs, in AI-F1, AI-F2, AI-F3, and C-sows. Our results indicated that although the size of EVs is similar between AI- and C-sows, the protein concentration of both oEVs and uEVs was significantly lower in AI-sows (*p* < 0.05). Proteomic analysis identified 38 unique proteins in oEVs from AI-sows, mainly involved in protein stabilization, glycolytic and carbohydrate processes. The uEVs from AI-sows showed the presence of 43 unique proteins, including already-known fertility-related proteins (EZR, HSPAA901, PDS). We also demonstrated that the protein composition of oEVs and uEVs differed depending on the seminal fraction(s) inseminated (F1, F2, or F3). In conclusion, we found specific protein cargo in oEVs and uEVs according to the type of semen fraction the sow was inseminated with and whose functions these specific EVs proteins are closely associated with reproductive processes.

## 1 Introduction

The ejaculation in boars is characterized by the release of semen in three visually differentiated fractions, each with specific color and consistency, associated with different cellular concentrations and biochemical compositions (Rodríguez-Martínez et al., 2009). The two main fractions of boar ejaculate are the sperm-rich fraction (SRF), which exhibits the highest concentration of sperm and accounts for 10%–30% of the total ejaculate volume and is well recognized by its dense white color, and the post-SRF, which represents the largest volume (70%–90% of the total ejaculate) but contains low levels of spermatozoa and has a watery aspect (Mann et al., 1981). The transition fraction between the SRF and the post-SRF, called intermediate fraction that is constituted by a higher volume than SRF, low concentration of spermatozoa, and greyish color. In farms, boar ejaculates are typically manually collected, retaining only the SRF to prepare seminal doses for artificial insemination (AI). The post-SRF and remaining seminal plasma (SP) are commonly discarded. However, in an increasing number of farms, the collection method is shifting towards semi-automated techniques, which enable the collection of the entire ejaculate and preservation of the large volume of SP present in the post-SRF (Aneas et al., 2008). It is well established that SP modulates sperm viability, function, and the ability to interact with the uterine epithelium and oocyte for successful fertilization (Rodríguez-Martínez et al., 2021). Moreover, the influence of SP extends beyond fertility, as its infusion into the uterus during the estrus persists throughout the preimplantation period, leading to modifications in the endometrial and embryonic transcriptome by upregulating genes and pathways related to maternal immune tolerance, embryonic development, implantation, and pregnancy progress (Martínez et al., 2019; Parrilla et al., 2020). SP triggers genetic and epigenetic pathways in spermatozoa that produce lasting changes in the female immune response with significant implications for progeny (Watkins et al., 2018; Morgan and Watkins, 2020). Importantly, studies have demonstrated that including all ejaculate fractions within seminal doses does not negatively impact reproductive performance regarding fertility, prolificacy, and animal growth (Luongo et al., 2022). As well as increasing the chances of the sperm reaching and fertilizing the egg, SP also has the potential to influence the development of the embryo (Martínez et al., 2020). This influence is thought to occur through specialized signaling pathways that interact with the female reproductive system.

Qualitative and quantitative differences in the SP proteome have been identified between the most relevant parts of boar ejaculate (Perez-Patiño et al., 2016), as well as in specific communication particles such as extracellular vesicles (EVs) (Barranco et al., 2019). EVs from SP play a regulatory role in female reproductive physiology in sows by modulating immune-related gene expression at the uterine level, facilitating spermatozoa fertilization of oocytes (Bai et al., 2018) and beyond. The reciprocal communication conceptus-endometrium is necessary for a successful pregnancy (Bazer and Johnson, 2014). This communication occurs through EVs released from the uterus (Kusama et al., 2018) and oviduct (Mazzarella et al., 2021), which are essential for regulating pivotal cellular activities during the peri-implantation period (Mittelbrunn and Sánchez-Madrid, 2012). The EVs released at uterine level modulate reproductive processes, such as follicular development in the ovary, oocyte maturation (Machtinger et al., 2016), maternal-embryonic communication (Almiñana et al., 2017) and the establishment of mammalian pregnancy. All of this may suggest a specific mechanism used by the uterine microenvironment to facilitate the fertilization process (Burns et al., 2014) and early embryonic development (Burns et al., 2016).

In relation to spermatozoa and once in the female genital tract, oviductal and uterine EVs (oEVs and uEVs, respectively) are transferred to the male gamete to ensure hyperactive sperm motility and fertilization potential (Nguyen et al., 2016). EVs originated from different parts of the female tract can be taken up by spermatozoa and influence their competition (Bridi et al., 2020). In addition, it has been demonstrated that incubation of sperm with endometrial cell-derived EVs could increase sperm tyrosine phosphorylation and the proportion of sperm undergoing the acrosome reaction (Franchi et al., 2016). Based on this finding, the exchange of EVs can be suggested as an emerging pathway by which cells of the female reproductive tract can interact with sperm (Murdica et al., 2020). The secretion of oEVs and uEVs has been demonstrated to be time-dependent and specific to the physiological status (reviewed by Bidarimath et al., 2021).

In this work, we hypothesized that SP from different accumulative ejaculate fractions might differentially affect the content of EVs in the oviductal fluid (OF) and uterine fluid (UF). Therefore, this study aimed to characterize sows’ oEVs and uEVs proteome in pregnant sows after AI with different accumulative fractions of the boar ejaculate and compare them to non-inseminated sows. The goal was to elucidate the potential effects of SP on reproductive events in the female reproductive tract.

## 2 Materials and methods

### 2.1 Reagents

All chemicals were obtained from Sigma-Aldrich Química, S.A. (Madrid, Spain) or Thermo Fisher Scientific (Waltham, MA, United States) unless otherwise indicated.

### 2.2 Animals

Fertile German Pietrain boars were housed in individual pens with sawdust, according to the European Commission Directive on the welfare of pigs, in the commercial farm Sergal Gestió Ramadera in Lleida (Spain). The temperature levels were automatically controlled by a system that kept constant the room temperature between 18°C and 22°C. Boars were fed with restricted diet according to their nutritional requirements. Water was provided *ad libitum*. Large White x Landrace crossbred sows (Danbred genetic) finalizing nursing were selected just after the weaning of their litters. The sows were selected according to specific criteria: similar body condition, age, and number of parities (between their third and fifth parity). After being separated from their litters, the selected sows were individually housed in gestation crates with unrestricted access to water and were provided with a daily feed allowance of 4.0 kg feed/day. Oestrus detection was performed from the day of weaning and once daily in the presence of a mature boar.

### 2.3 Semen collection

Ejaculates from boars with proven fertility were collected using the manual method by an experienced technician. The sample was separated at the time of collection by the visual perception of the different seminal fractions of the ejaculate based on their volume, color, and consistency: i) the SRF, identified by its characteristic dense white color; ii) the intermediate fraction, which was characterized by larger volume than SRF and moderate dense white color; iii) the post-SRF, characterized by a watery liquid appearance due to the absence or very low number of spermatozoa. The pre-SRF and the gel fraction were discarded. Samples from each boar and seminal fraction were evaluated microscopically for sperm concentration, motility, acrosome integrity, and normal morphology using standard laboratory techniques and performed by experienced technicians to meet normal standards of seminal samples for AI under commercial requirements.

### 2.4 Preparation of semen doses and artificial insemination (AI)

Immediately after the collection of each ejaculate fraction, the samples were processed to obtain AI doses with a total final volume of 60 mL. The sperm concentration in the samples was first determined using a calibrated sperm analyzer (Androvision^®^ Minitüb, Tiefenbach, Germany) and then diluted in the AndroStar^®^ Plus extender (Minitüb, Tiefenbach, Germany) to achieve a final concentration of 33 × 10^6^ spermatozoa/mL (2 × 10^6^ total spermatozoa/dose 60 mL). Sows were inseminated in individual stalls using a post-cervical AI method at the onset of estrus and 24 h later. The AI was performed with the combined catheter-cannula kit Soft & Quick^®^ (Tecno-Vet, S.L., Barcelona, Spain), which was inserted to the uterine body by an experienced technician according to the standard protocol for post-cervical AI commonly used in pig farms.

### 2.5 Fluids collection

Female reproductive tracts from sows were collected at the local abattoir and transported to the laboratory within 60 min. Once in the laboratory, the uterine tracts were washed twice in physiological saline (0.9% NaCl) supplemented with 0.1% antibiotic kanamycin. Tissue dissection was performed on a cooled surface, keeping the uterus and oviducts together from the same animal.

#### 2.5.1 Oviductal fluid (OF) collection

The OF collection was performed as previously described by Kusama et al. (2018). Briefly, the oviductal lumen of the two oviducts from the same animal was flushed in the direction from the ampulla to the isthmus with 5 mL phosphate-buffered saline (PBS) at 4°C and using a catheter (24G BD Insyte™, 381212, Becton Dickinson Infusion Therapy Systems, Inc., Sandy, Utah, United States) adapted to a 10 mL syringe. The fluid collected after the oviduct flushing with PBS was processed to isolate EVs.

#### 2.5.2 Uterine fluid (UF) collection

For the collection of UF, the uterus was irrigated in the direction from the caudal uterine horn to the utero-tubal junction with 10 mL PBS at 4°C using a 10 mL syringe. Immediately after collection, the flushing medium from both uterine horns was placed into Petri dishes and embryos were isolated from the obtained flushes under stereomicroscope to ensure pregnancy and were assessed for morphological quality and developmental stage classified as morula or blastocyst. The fluid collected after the uterus flushing with PBS was collected and processed to isolate EVs.

### 2.6 Isolation and characterization of extracellular vesicles (EVs)

EVs were isolated from oviductal and uterine flushings according to the protocol of serial ultracentrifugation described by Théry et al. (2006) and Almiñana et al. (2017) with some modifications. Briefly, each experimental group’s flushings from the oviducts and uteri were centrifuged at 300 × *g* for 15 min at 4°C to remove epithelial cells. The supernatant was collected and transferred to a new tube, then centrifuged at 2,000 × *g* for 10 min at 4°C to remove cellular debris. The supernatant was then ultracentrifuged at 100,000 × *g* for 70 min at 4°C (Beckman Coulter Optima L-100 XP ultracentrifuge with 70ti rotor) to pellet the EVs. The supernatant was discarded, and the pellet was resuspended in 3.5 mL PBS and ultracentrifuged again under the same conditions. The pellet was resuspended in a final volume of 300 µL PBS and aliquoted to 100 µL. One aliquot of EVs suspensions was analyzed fresh for protein concentration using the Coomassie Plus Bradford assay kit (23238, Fisher Scientific™, Waltham, MA, United States of America) according to the manufacturer’s protocol. The same amount of protein was processed and analyzed in each sample within the same type of reproductive fluid, OF or UF, corresponding to the sample with the lowest total amount of protein. The rest of the aliquots were stored at −80°C until further analysis.

#### 2.6.1 EVs morphology

The presence and morphology of EVs were determined by Transmission Electron Microscopy (TEM). Samples were processed according to the protocol described by Théry et al. (2006) with the following modifications. A 10 µL aliquot of the vesicle suspension was placed on a Formvar-Carbon-Coated grid for 30 s at room temperature. The vesicle-coated grids were washed once with distilled water for 1 min and then stained with 10 µL of 2% uranyl acetate for 1 min for negative contrast. The samples were air-dried at room temperature for 20 min. Photographs were taken using a JEOL1011 electron microscope slide at 80 kV (Jeol, Japan). Digital images were taken at 59,000–97,000 magnification.

#### 2.6.2 EVs size

The size distribution of EVs was measured by Dynamic Light Scattering (DLS) according to Sabín et al. (2007) and using a Malvern Autosizer 4,800 (Malvern Instruments, Malvern, United Kingdom) equipped with a solid state He-Ne laser at a wavelength of 488 nm. The intensity of the scattered light was measured at 25°C. A 10-μL aliquot of the vesicle suspension was diluted up to 1 mL in PBS and transferred to a disposable solvent-resistant cuvette specific for the DLS analysis. Data were acquired and analyzed using the PCS software (version 1.61, Rev. 1, Malvern Instruments, Malvern, United Kingdom) in the automatic acquisition mode. We chose to use the intensity values as they best represent the data of the pure sample, as they assume the least error (criterion taken from Malvern Instruments INC). In addition, particle intensity classification provides more accurate data on the size of the EVs.

#### 2.6.3 EVs quantitation

EVs quantitation was performed using the EXOCET Exosome Quantitation Kit (System Biosciences, SBI) which is an enzymatic, colorimetric assay designed as a direct measurement of esterase activity known to be within exosomes.

Fresh EVs pooled pellets in a concentrated solution (corresponding to a protein concentration of 1–2 μg/μL) were resuspended with kit lysis Buffer at (1:4, v:v) to a total volume of 100 μL per reaction. EVs lysates were incubated at 37°C for 5 min and centrifuged at 1,500 *g* for 5 min to remove cell debris. 50 μL of transferred supernatants and standards were added to clear microtiter wells and mixed with 50 μL of reaction buffer (buffer A + buffer B) up to a total 100 µL volume in a 96 well plate (Nunclon Delta, Thermo Scientific). Replicates of each sample were made fourfold, and the microtiter plate was incubated for 20 min at room temperature. Optical density was read using a spectrophotometric plate reader (Apollo 11 LB913, Berthold Technologies GmbH & Co., TN, United States) at 405 nm. Finally, quantitate results were obtained by calculating the standard curve, previously calibrated by Nano Sight analysis, and plotting the sample readings on the standard curve. Quantitative results were represented in number of particles per mL.

#### 2.6.4 EVs proteins immunoblotting

Protein extraction from 50 μL of the EVs suspension was carried out adding RIPA Lysis Buffer System (Santa Cruz Biotechnology Inc., Dallas, TX, United States) at 1:1 (v:v). The mixtures were pipetted up and down to resuspend the pellet for 5 min, incubated 30 min on ice, placed in an ice-cold sonication bath for 10 s at 20% amplitude and were centrifuged at 14,000 *g* for 5 min at 4°C to remove cell debris (Subedi et al., 2019). The supernatants were transferred to new tubes and the protein concentration was determined using the Bradford protein assay (Bio-Rad Laboratories, Inc., Hercules, CA, United States).

Equal protein amount of each sample (40 µg) was mixed with reducing Laemmli-buffer and was resolved on 10% sodium dodecyl sulphate polyacrylamide gel electrophoresis (10% Mini-PROTEAN^®^ TGX™ Precast Protein Gels, Bio Rad^®^) and transferred to a 0.45 µm polyvinylidene difluoride nitrocellulose (PVDF) membrane (Immobilon®-P Transfer Membranes). Membranes were washed with distilled water and unspecific unions were blocked with Tris buffered saline containing 0.1% Tween 20 (T-TBS; P-1379, Sigma-Aldrich^®^, Madrid, Spain) supplemented with 5% skimmed milk for 1 h at room temperature. After that, membranes were incubated with the primary antibodies anti-HSP70 (Sigma Aldrich; Cat # H5147-2ML, 1:1.000) and anti-CD63 (Santa Cruz Biotechnology; Cat # sc-5275, 1:100) in blocking solution overnight at 4°C. After washing with fresh T-TBS, membranes were incubated for 1 h in goat anti-mouse IgG secondary antibody conjugated to horseradish peroxidase (HRP) in blocking solution (Du et al., 2016). Positive immunoreactive bands were detected by an enhanced chemiluminescence (ECL) substrate (Thermo Fisher Scientific, Waltham, MA, United States) and chemiluminescence was detected with Amersham™ Imager 600 (GE Healthcare) equipment.

### 2.7 Proteomic analysis of extracellular vesicles (EVs)

#### 2.7.1 In-solution trypsin digestion

The EVs suspensions were thawed at 4°C and subsequently subjected to digestion. This digestion process took place in a solution containing 100 µL of a buffer consisting of 50 mM ammonium bicarbonate with a pH of 8.5. To aid in the digestion, a small amount (0.01%, v:v) of ProteaseMax (Promega, WI, United States) was added. In addition, the samples were reduced by introducing 20 mM of dithiothreitol (DTT) and incubating the mixture for 20 min at 56°C. Following this, the samples were alkylated by adding 100 mM of iodoacetic acid (IAA) and allowing it to react for 30 min at room temperature while keeping the environment dark. Once the alkylation was completed, the digestion process was initiated by adding Trypsin Gold Proteomics Grade (Promega) to the mixture at a ratio of 1:100 (w/w). The digestion was carried out for a duration of 3 h at 37 °C. To halt the reaction, 0.1% formic acid was added, and the resulting mixture was filtered through a filter with a pore diameter of 0.2 µm. Finally, the samples were dried using an Eppendorf Vacuum Concentrator 5301.

#### 2.7.2 High-performance liquid chromatography-mass spectrometry analysis (HPLC-MS/MS analysis)

The separation and analysis of the digested peptides from the samples were performed using an HPLC/MS system. This system consisted of an Agilent 1290 Infinity II Series HPLC (Agilent Technologies, Santa Clara, CA, United States) equipped with an Automated Multisampler module and a High-Speed Binary Pump. The HPLC system was connected to an Agilent 6550 Q-TOF Mass Spectrometer, and the interface used was the Agilent Jet Stream Dual electrospray (AJS-Dual ESI). The experimental parameters for both the HPLC and Q-TOF components were configured in the MassHunter Workstation Data Acquisition software (Agilent Technologies, Rev. B.08.00).

To prepare the dry samples resulting from trypsin digestion, they were reconstituted in a 20 µL volume of resuspending buffer. This buffer, known as buffer A, was composed of a mixture of water, acetonitrile, and formic acid in the ratio of 94.9:5:0.1, respectively. The reconstituted samples were injected into an Agilent AdvanceBio Peptide Mapping HPLC column with dimensions of 2.7 µm × 100 × 2.1 mm, which was thermostatted at 50°C. The injection of the samples occurred at a flow rate of 0.4 mL/min. Subsequently, the column was washed with buffer A for a duration of 3 min, and the digested peptides were eluted using a linear gradient of buffer B (acetonitrile:water:formic acid, 97:2.9:0.1). This gradient started at 0% and gradually increased to 40% over a period of 40 min. After that, there was a linear gradient from 40% to 95% of buffer B for 8 min, followed by a 95% concentration of buffer B for 3 min. Finally, the column was equilibrated in the initial conditions for 6 min before each subsequent injection. Ten µg of albumin were injected to check the purity of the samples.

The mass spectrometer was operated in the positive mode. The nebulizer gas pressure was set at 35 psi, while the drying gas flow rate was maintained at 14 L/min at a temperature of 300°C. The sheath gas flow rate was set at 11 L/min with a temperature of 250°C. The capillary spray, nozzle, fragmentor, and octopole RF Vpp voltages were configured at 3500 V, 100 V, 360 V, and 750 V, respectively. Profile data were collected for both MS and MS/MS scans using the extended dynamic range mode at a scan rate of 4 GHz. The mass range for both MS and MS/MS scans was set between 50–1700 m/z, and the scan rates were 8 spectra/sec for MS and 3 spectra/sec for MS/MS. Auto MS/MS mode was used, and precursor selection was based on abundance with a maximum of 20 precursors selected per cycle. A ramped collision energy was employed with a slope of 3.68 and an offset of −4.28. The exclusion of the same ion was applied after two consecutive spectra.

The data processing and analysis were conducted using the Spectrum Mill MS Proteomics Workbench software (Rev B.06.00.201, Agilent Technologies, Santa Clara, CA, United States). The raw data were extracted using default conditions, which included the identification of unmodified or carbamidomethylated cysteines, a range of [MH]+ 50–10,000 m/z, a maximum precursor charge of +5, and a minimum signal-to-noise MS (S/N) ratio of 25. The software also utilized specific parameters for peak finding, such as the identification of 12C signals.

For the MS/MS search, an appropriate and updated protein database was employed, and the search criteria included variable modifications (carbamidomethylated cysteines, STY phosphorylation, oxidized methionine, and N-terminal glutamine conversion to pyroglutamic acid), a tryptic digestion allowing for up to 5 missed cleavages, the use of an ESI-Q-TOF instrument, a minimum matched peak intensity of 50%, a maximum ambiguous precursor charge of +5, monoisotopic masses, a peptide precursor mass tolerance of 20 ppm, a product ion mass tolerance of 50 ppm, and the calculation of reversed database scores. The validation of peptide and protein data was performed using auto thresholds.

### 2.8 Experimental design

**FIGURE 1:**
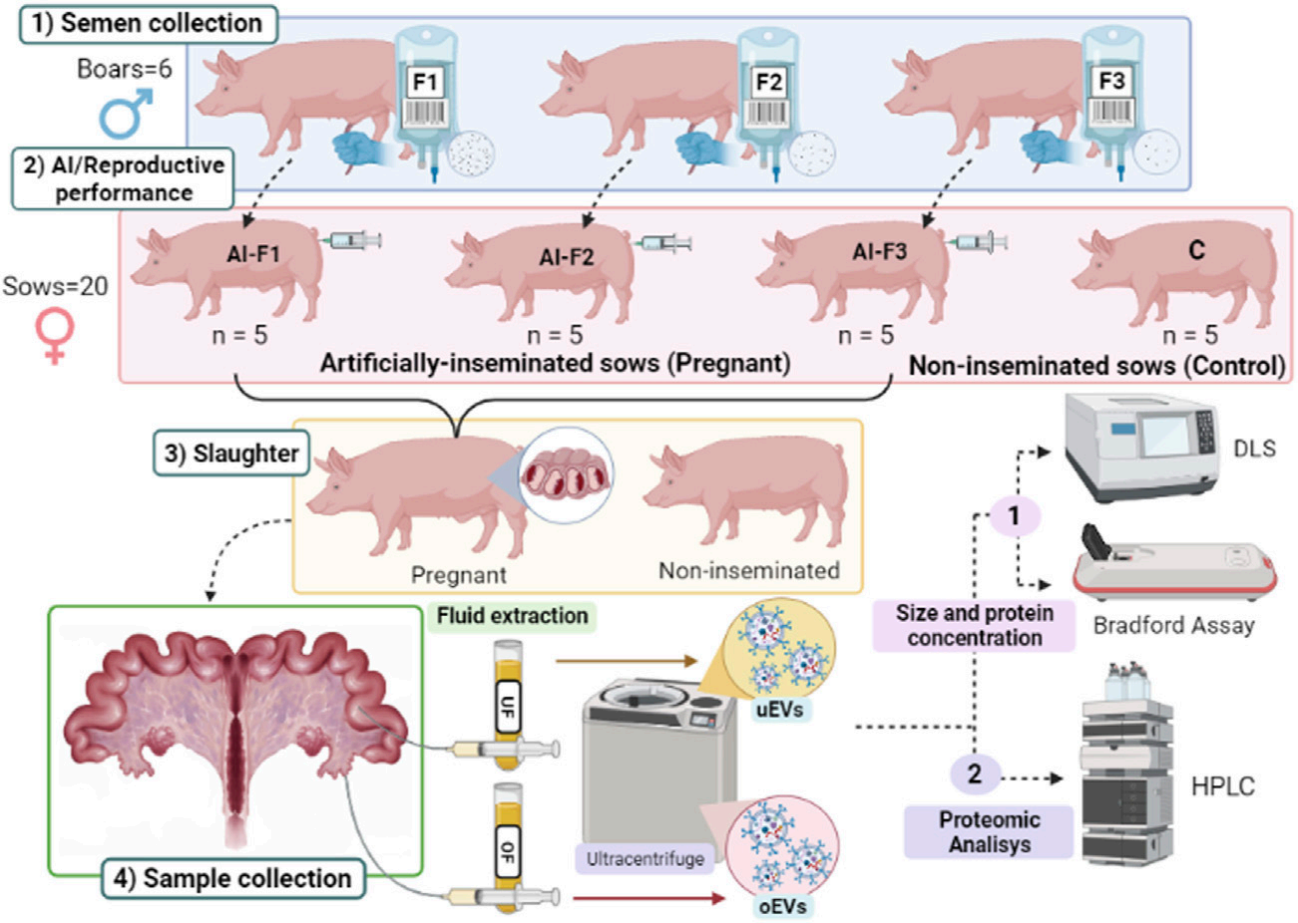
Diagram of the experimental design. 1) Semen samples were collected from six different boars and prepared as three types of ejaculate accumulative fractions: F1, composed of the rich-sperm fraction (SRF); F2, composed of the F1 plus the intermediate fraction; and F3, composed of F2 plus the post-SRF. 2) Artificial insemination (AI) was performed: Four experimental groups of sows (*n* = 5 sows/group) were inseminated with a type of AI dose (AI-F1, AI-F2, and AI-F3) or non-inseminated (C). 3) Sows were slaughtered on day six post-AI, and female genital tracts were collected and identified according to the experimental group they belonged. 4) Sampling of the female genital tracts: Oviductal and uterine flushing’s were extracted for the isolation of extracellular vesicles (EVs) by ultracentrifugation, characterization analysis of the size distribution by dynamic light scattering, and determination of the protein concentration by the Bradford assay (protein concentration). The protein quantification of EVs from the oviductal fluid (oEVs) and uterine fluid (uEVs) were evaluated by HPLC/MS-MS.

The semen samples were collected from six boars, and the AI doses were composed according to the study performed by Luongo et al. (2022). Briefly, three different types of AI doses were produced based on their appearance and composition in ejaculate fractions i) F1, composed of the SRF; ii) F2, composed of F1 plus the intermediate fraction; iii) F3, composed of F2 plus the post-SRF. A grand total of 20 female pigs (sows) were allocated randomly into four separate groups, with each group receiving a distinct type of AI dose (AI-F1, AI-F2, or AI-F3) or non-inseminated (control, C). On day six post-AI, all sows were sacrificed, and their uterine tracts were collected and identified according to the experimental group they belonged to. Each genital tract was dissected to obtain OF and UF for EVs’ isolation, characterization, and proteomic analysis. Note that all AI-sows were pregnant at the time of slaughter. Figure 1 depicts a visual representation of the experimental design.

### 2.9 Bioinformatic analysis–Annotation of human homologs and gene ontology analysis

Raw Uniprot IDs obtained from the proteomics results were utilized to identify the most extensively annotated IDs in *Sus scrofa* and their corresponding homologs in the human species. This was achieved by querying the UniProt API (UniProt Consortium, 2018) using a custom Python 2.7 script. Subsequently, the UniProt IDs were annotated using the functional annotation tool of the Database for Annotation, Visualization, and Integrated Discovery (DAVID, version 6.8) (Bateman, 2019). The Gene Ontology (GO) includes three orthogonal ontologies: biological process (BP), molecular function (MF), and cellular component (CC). To eliminate redundant terms, the most statistically significant GO terms (FDR <5%) were assessed using REVIGO (Supek et al., 2011). The selected GO terms were then visualized by plotting the corresponding percentages using a custom R script incorporating the dplyr (Wickham et al., 2019) and ggplot2 (Wickham H., 2016) libraries. Additionally, a functional clustering analysis of the proteins present in extracellular vesicles (EVs) from each fluid was performed using the DAVID functional clustering tool. Venn diagrams comparing the five experimental groups were generated using Venn diagrams at https://bioinformatics.psb.ugent.be/webtools/Venn/.

### 2.10 Statistical analysis

All statistical analyses were performed using IBM SPSS 24.0 software package (SPSS Inc. Chicago, IL, United States). EVs quantitation, protein concentration, and size are presented as the mean ± standard error of the mean (SEM). The variables in all experiments were tested for their normality by Shapiro-Wilk and homogeneity of variances before analysis by two-way ANOVA considering the group of sows (AI-F1, AI-F2, AI-F3, and C) and pregnancy as factors, followed by a *post hoc* Tukey test. The non-parametric Kruskal Wallis test was used for the variables whose data were not normally distributed. Differences between treatments were considered statistically significant at *p* < 0.05.

## 3 Results

### 3.1 Characterization of EVs from the OF and UF

#### 3.1.1 Presence of EVs and characterization by transmission electron microscopy (TEM)

**FIGURE 2:**
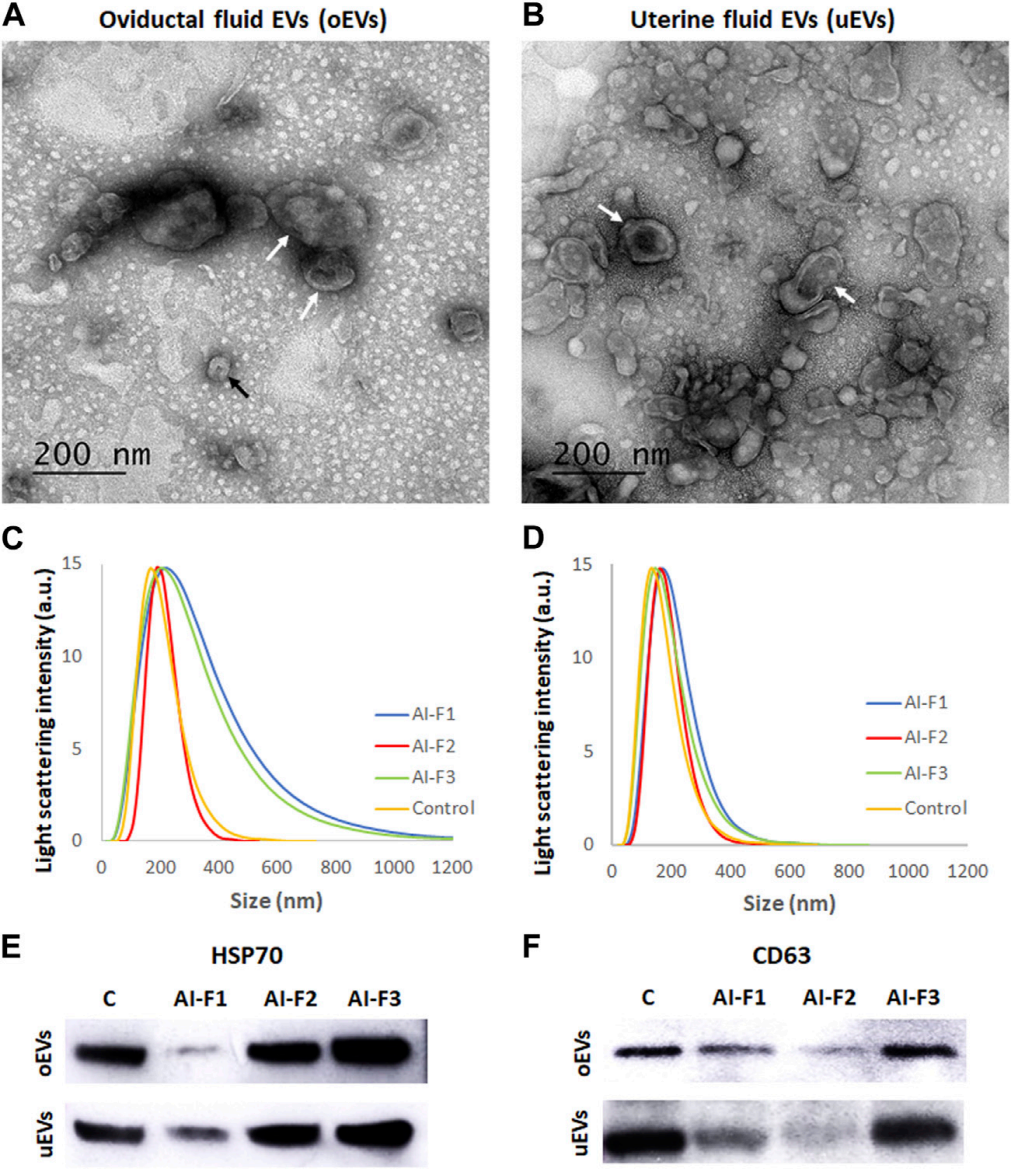
Characterization the extracellular vesicles’ size from oviductal fluid (oEVs) and uterine fluid (uEVs). **(A)** Transmission electron microscopy (TEM) of oEVs after the OF ultracentrifugation. **(B)** Transmission electron microscopy (TEM) of uEVs after the UF ultracentrifugation. Concentration of uEVs was higher than oEVs, independently of the type of AI-dose inseminated (F1, F2 or F3). Arrows indicate the cup-shaped morphology of EVs. **(C)** Graphical distribution of the oEVs by size and sow depending on the type of AI-dose inseminated (AI-F1, AI-F2, or AI-F3) or non-inseminated (control, C). **(D)** uEVs populations by size and sow depending on the experimental group (AI-F1, AI-F2, AI-F3, or C). **(E)** EVs protein marker HSP70 in the experimental groups (AI-F1, AI-F2, AI-F3, or C). **(F)** EVs protein marker CD63 in the experimental groups (AI-F1, AI-F2, AI-F3, or C).

TEM observations confirmed the presence of EVs in porcine oviductal and uterine flushings (Figures 2A, B). The presence of two populations of EVs in the fluid was observed as two different peaks detected in sows from the four experimental groups, either in OF or UF (Figures 2C, D). Diverse populations of EVs ranging in size (142.40–297.76 nm) were observed. These EVs appeared as round or cup-shaped membrane-surrounded vesicles in their native stage. We observed a population of small EVs (30–150 nm) resembling exosomes and a population of large EVs (>150 nm) resembling microvesicles. Very large EVs (>1,000 nm) were considered as aggregates of multiple EVs.

The size of the oEVs and uEVs was analyzed and compared and there were no statistically significant differences between groups of sows (*p* > 0.05; Table 1). In general terms, oEVs tended to be larger (ranging from 215.52 to 297.76 nm) than uEVs (ranging from 142.840 to 202.92 nm); however, there were no differences in size between groups of sows (Table 1).

**TABLE 1:** EVs’ size, quantitation, and protein concentration in the oviductal fluid (oEVs) and uterine fluid (uEVs) from artificially inseminated sows with three different types of accumulative fractions of the boar ejaculate: AI-F1 (*n* = 5), AI-F2 (*n* = 5), and AI-F3 (*n* = 5); and from non-inseminated sows: C (*n* = 5). F1, sperm-rich fraction (SRF); F2, F1 plus the intermediate fraction; F3, F2 plus the post-SRF.

		AI-F1	AI-F2	AI-F3	C
Size (nm)	oEVs	297.76 ± 89.45	262.60 ± 98.57	234.84 ± 5.67	215.52 ± 9.65
	uEVs	142.40 ± 30.53	202.92 ± 18.20	176.86 ± 8.45	163.86 ± 12.45
[EVs] (x 10^9^ particles/mL)	oEVs	278.36 ± 10.32	392.28 ± 20.79	404.07 ± 26.29	414.79 ± 106.53
	uEVs	321.92 ± 8.05^a^	395.14 ± 32.00^a^	525.14 ± 32.81^ab^	687.28 ± 85.19^b^
[Protein] (µg/µL)	oEVs	0.51 ± 0.07	0.55 ± 0.05	0.53 ± 0.06	0.58 ± 0.05
	uEVs	0.86 ± 0.06^a^	0.86 ± 0.04^a^	1.19 ± 0.09^b^	1.30 ± 0.06^b^

Data are represented as the mean ± standard error of the mean (SEM). Different superscripts (^a^,^b^) within the same row indicate statistical differences between experimental groups (*p* < 0.05).

The immunoblotting revealed that EVs were present both in the OF and UF of all sows in study (Figure 2) since EVs protein markers HSP70 (2E) and CD63 (2F) were detected in AI- and C-sows.

#### 3.1.2 EVs quantitation and protein concentration

The results of the quantitation of oEVs particles were similar for all experimental groups (Table 1), ranging from 278.36 to 414.79 × 10^9^ particles/mL (*p* > 0.05). However, the concentration of uEVs particles were lower (*p* < 0.05) in AI-F1 and AI-F2 (321.92 ± 8.05 and 395.14 ± 32.00 × 10^9^ particles/mL) than in C (687.28 ± 85.19 × 10^9^ particles/mL). AI-F3 showed an intermediate concentration of uEVs particles with respect to the rest of groups (525.14 ± 32.81 × 10^9^ particles/mL; *p* > 0.05).

The oEVs and uEVs protein concentration (protein cargo) was also compared in AI- and C-sows (Table 1). No statistical differences were found in the protein cargo of the total intact oEVs, ranging from 0.51 to 0.58 μg/μL (*p* > 0.05). In uEVs, the protein cargo of AI-F1 and AI-F2 (0.86 ± 0.06 and 0.86 ± 0.04 μg/μL) were significantly lower than AI-F3 and C (1.19 ± 0.09 and 1.30 ± 0.06 μg/μL).

### 3.2 Proteomic analysis

#### 3.2.1 Proteins identified in EVs from oviductal (oEVs) and uterine fluids (uEVs)

Initially, the purity of the samples was confirmed since the samples for all the experimental groups only showed between 0.00% and 7.61% of the albumin’s spectra and between 0.00% and 0.66% of the albumin’s intensity. A total of 645 proteins were identified in oEVs and uEVs. To minimize the risk of false positives, only proteins with a minimum of two peptides and present in at least three of the five replicates were considered, resulting in 362 final proteins. The list of proteins identified in the different accumulative ejaculate fractions, including the relative number of peptides of each protein, is shown in Supplementary Tables S1–4 (Supplementary File S1) and Supplementary File S2.

Interestingly, a comparison of the oEVs and uEVs proteome of AI- and C-sows revealed four proteins common to all groups: 15S Mg (2^+^)-ATPase p97 subunit, annexin (ANXA), glyceraldehyde-3-phosphate dehydrogenase (GAPDH) and tubulin alpha 1 chain (TUBA1C). These four proteins are involved in calcium signaling, tissue remodeling, immune modulation, and antioxidant defense systems. They exert biological functions that are regulated in the fallopian tube and endometrium. We decided to compare the oEVs and uEVs proteins from AI- and C-sows since it provides valuable insights into the molecular mechanisms involved in reproductive events and potentially influences the outcome of offspring development. The nature of the proteins and the possible differences are described below.

#### 3.2.2 Protein characterization of oEVs

**FIGURE 3:**
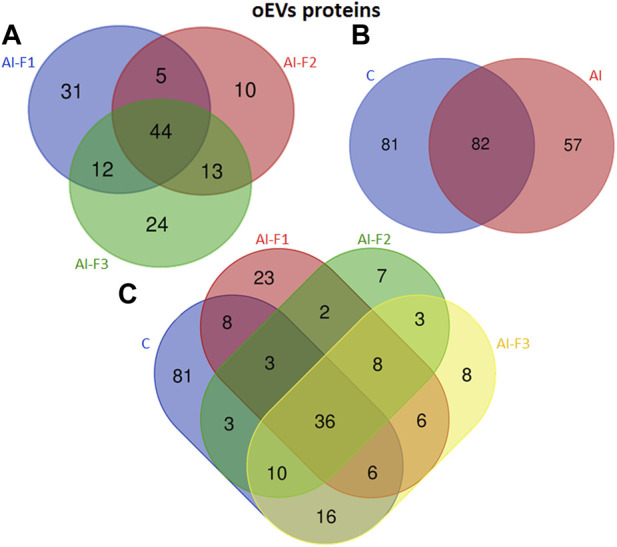
Venn diagram illustrating the number of different proteins from extracellular vesicles of oviductal fluid (oEVs) in sows (*n* = 20) inseminated with different semen doses from boars (*n* = 6) according to the seminal fraction(s) used for artificial insemination (AI): F1, composed by the sperm-rich fraction (SRF); F2, composed by F1 plus the intermediate fraction; F3, composed by F2 plus the post-SRF. Non-inseminated sows were considered as control (C). **(A)** The overlap of oEVs proteins from AI-F1, AI-F2, and AI-F3 sows. **(B)** The overlap of oEVs proteins of AI- and C-sows. **(C)** The overlap of oEVs protein in all the experimental groups.

A total of 220 proteins were detected across all experimental groups. Figure 3 shows specific proteins from each experimental group and the common ones in two or more experimental groups. In addition, all proteins from oEVs are listed in Supplementary Tables S1, S2 (Supplementary File S1). A total of 139 proteins were detected in oEVs from AI-sows and 92 of them were detected in oEVs from AI-F1 group 31 of which were exclusive to this group. A total of 72 proteins were detected in oEVs from AI-F2 sows, 10 of which were exclusive to this group. Finally, 93 proteins were detected in oEVs from AI-F3 sows, 24 of which were exclusive to this group. In addition, the comparison between the three experimental groups identified 44 common proteins (Figure 3A). When C-sows were compared with AI-sows, 82 proteins were common to both groups. Of these, 57 proteins were exclusive to AI-sows and 81 proteins were exclusive to C-sows (Figure 3B).

The number and comparative overlap of the identified oEVs proteins in AI- and C-sows are illustrated in Figure 3C and listed in Supplementary Tables S1, S2 (Supplementary File S1). Eighty-one of the 220 proteins were exclusively present in C-sows: 14-3-3 protein theta (YWHAQ), cadherin 13 (CDH13), galectin-3 binding protein (LGALS3), and ubiquitin C (UBC). Twenty-three proteins were characteristically exclusive of AI-F1 sows: 14-3-3 protein zeta/delta (YWHAZ), 6 phosphogluconate dehydrogenase decarboxylating (PGD), peptidyl-prolyl trans isomerase E (PPIE), and tubulin alpha-1A chain (TUBA1A). In addition, 7 proteins were exclusive of AI-F2 sows: GTP-binding nuclear protein Ran (RAN), NIMA-related kinase 1 (NEK1), peroxiredoxin-2 (PRDX2), and syndecan-binding protein (SDCBP). Finally, 8 proteins were found exclusively in AI-F3 sows: Heat shock protein 70 kDa 1B (HSPA1B), haemoglobin subunit epsilon (HBE), profilin (PFN1), and tyrosine 3-monooxygenase/tryptophan 5-monooxygenase activation protein zeta (YWHAZ).

#### 3.2.3 Functional analysis of oEVs proteins

**FIGURE 4:**
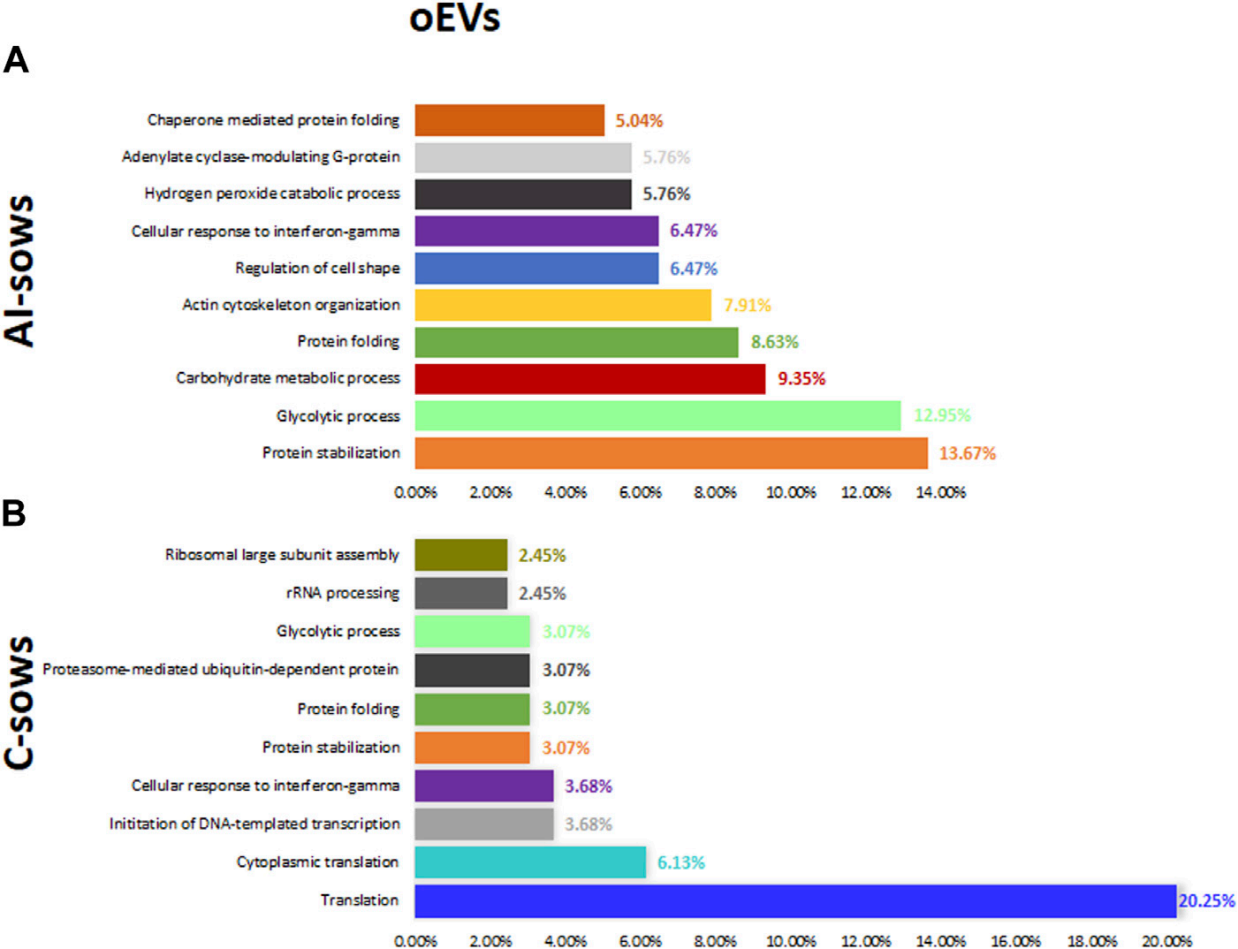
Biological processes and the percentage of extracellular vesicles detected in oviductal fluid (oEVs) in **(A)** artificially inseminated sows (AI-sows) and **(B)** non-inseminated sows (control, C-sows).

GO includes three orthogonal ontologies: biological process (BP), molecular function (MF), and cellular component (CC), although we only focused on BP, which is more relevant in our study. Identification of enriched pathways using GO analysis revealed 45 pathways related to oEVs proteins. In Figure 4, we have presented the results of the GO analysis, focusing on the ten most enriched pathways in artificially inseminated sows and non-inseminated control sows. All the enriched pathways involved in biological processes are listed in Supplementary File S3. The top 5 biological processes in F1 group included pathways involved in protein stabilization, protein folding, glycolytic process, proteasome-mediated ubiquitin-dependent protein catabolic process and positive regulation of RNA polymerase II transcriptional preinitiation complex assembly. We also identified enriched pathways in the F2 group that are involved in glycolytic process, vesicle-mediated transport, protein stabilization, protein folding and regulation of cell shape. However, the most enriched pathways in the sows inseminated with F3 were translation, protein stabilization, innate immune response, glycolytic process and DNA-templated transcription and initiation.

#### 3.2.4 Protein characterization of uEVs

**FIGURE 5:**
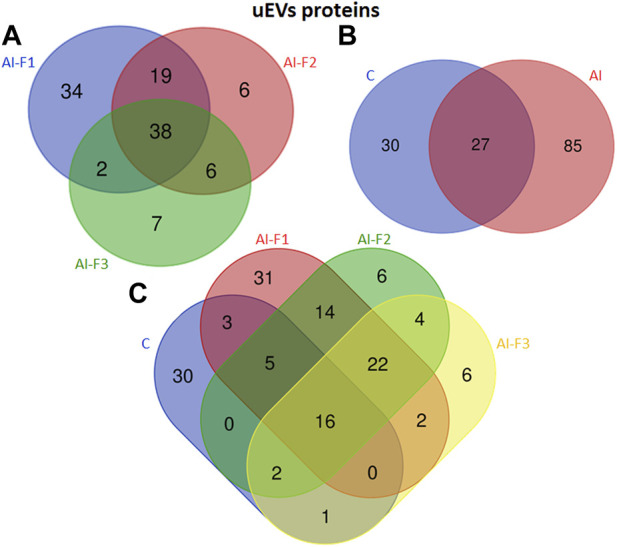
Venn diagram illustrating the number of different proteins from extracellular vesicles of uterine fluid (uEVs) in sows (*n* = 20) inseminated with different semen doses from boars (*n* = 6) according to the seminal fraction(s) used for artificial insemination (AI): F1, composed by the sperm-rich fraction (SRF); F2, composed by F1 plus the intermediate fraction; F3, composed by F2 plus the post-SRF. Non-inseminated sows were considered as control (C). **(A)** The overlap of uEVs proteins from AI-F1, AI-F2, and AI-F3 sows. **(B)** The overlap of uEVs proteins of AI- and non-inseminated sows (control, C). **(C)** The overlap of uEVs protein in all the experimental groups.

A total of 142 proteins were identified in uEVs. Figure 5 illustrates the exclusive proteins found in each group, as well as the shared proteins among the different experimental groups. The detailed lists of exclusive and common proteins can be found in Supplementary Tables S3, S4 (Supplementary File S1), respectively.

A group of 112 proteins were detected in uEVs proteins from AI-sows. Ninety-three proteins were detected in AI-F1 sows, being 34 of them, exclusive to this group. In uEVs from AI-F2 sows, a total of 69 proteins were detected, being 6 of them, exclusive to this group. Fifty-three proteins were detected in uEVs from AI-F3 sows, being 7 of them exclusive to this group.

The analysis of uEVs detected 38 proteins present in the three groups of AI-sows. In addition, 19 proteins were common in AI-F1 and AI-F2 sows, two proteins between AI-F1 and AI-F3 sows, and six proteins between AI-F2 and AI-F3 sows (Figure 5A). Comparing AI- with C-sows (Figure 5B), 85 proteins were exclusive to AI-sows, 30 to C-sows, and 27 were shared by the two groups.

The number and comparative overlap of the identified oEVs proteins in AI- and C-sows are illustrated in Figure 5C and listed in Supplementary Tables S3, S4 (Supplementary File S1). Thirty of the 142 proteins were characteristically exclusive of C-sows and included beta-2-microglobulin (B2M), clathrin heavy chain (CLTC), heat shock protein family A member 8 (HSPA8), and vitronectin (VTN). Thirty-one proteins were characteristically exclusive of AI-F1 sows, including 14-3-3 domain-containing protein (YWHAG), calmodulin-3 (CALM3), ezrin (EZR), and heat shock protein HSP90-alpha (HSP90AA1). In addition, six proteins were exclusive to AI-F2 sows: 14-3-3 protein zeta/delta (YWHAZ), adenylyl cyclase-associated protein (CAP1), heterogeneous nuclear ribonucleoprotein K (HNRNPK), and Na^+^-dependent phosphate cotransporter 2B (NPT2B). Finally, six proteins were found exclusively in AI-F3 sows: ATP-binding cassette subfamily G member 2 (ABCG2), pendrin (SLC26A4), proline-rich transmembrane protein 1B (PRRT1B), and STEAP family member 4 (STEAP4).

#### 3.2.5 Functional analysis of uEVs proteins

**FIGURE 6:**
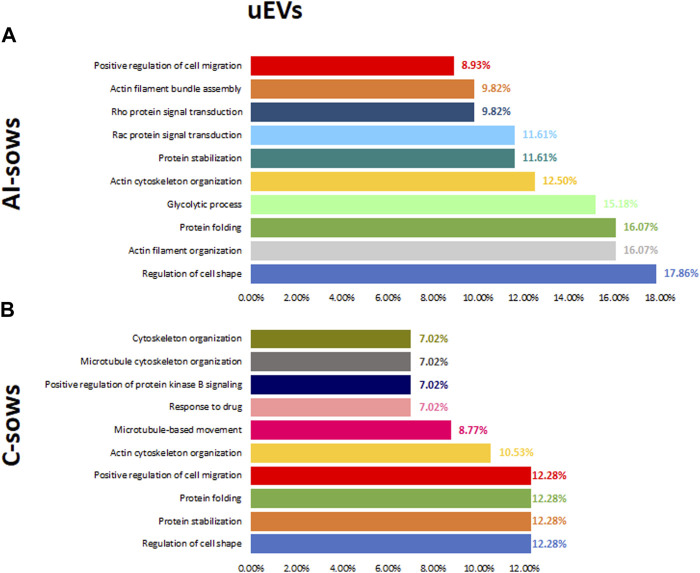
Biological processes and the percentage of extracellular vesicles detected in uterine fluid (uEVs) in **(A)** artificially inseminated sows (AI-sows) and **(B)** non-inseminated sows (control, C-sows).

Identification of enriched pathways using GO analysis revealed 62 pathways in uEVs proteins. In Figure 6, we have presented the results of the GO analysis, focusing on the ten most enriched pathways in artificially inseminated sows and non-inseminated control sows. All the enriched pathways involved in biological processes are listed in (Supplementary File S4). The top 5 biological processes in F1 included protein stabilization, protein folding, glycolytic process, regulation of cell shape and protein localization to plasma membrane. The enriched pathways in F2 were also related to regulation of cell shape, protein folding, actin filament organization, protein stabilization and small GTPase mediated signal transduction. We also identified enriched pathways in the F3 group that are involved in regulation of cell shape, glycolytic process, actin filament organization, actin cytoskeleton organization and sensory perception of sound.

## 4 Discussion

The present study aimed to gain insight into the changes in EVs protein cargo in non-inseminated and pregnant sows artificially inseminated with three accumulative fractions of the boar ejaculate. The objective was to elucidate how the SP from different ejaculate fractions might influence the oviductal and endometrial EVs proteome. The oviduct and uterus of pregnant sows undergo significant physiological and biochemical changes to support the development and growth of the conceptus. One of these changes is a modification in the quantity and diversity of proteins present. However, the amount of protein in the uEVs cargo in pregnant and non-pregnant sows has not been determined yet.

Contrary to expectations, our results revealed that the protein concentration in EVs from the OF and UF was higher in non-pregnant compared to pregnant sows. An explanation for this result could be attributed to the analyzed day of gestation. We determined the protein cargo on day 6 of pregnancy, while maternal recognition typically occurs around day 11–12 (Bazer et al., 1997). This hypothesis is supported by the results obtained by Rudolf Vegas and co-workers (Rudolf Vegas et al., 2022), who observed that the EVs protein cargo of UF collected from pregnant mares was similar to that of cyclic control mares on day 10 of gestation, with an increase in protein concentration occurring later. Hu et al. (2022) obtained similar results in pigs on day 10 of gestation. It seems that the type of protein is likely more important than the amount of protein in early pregnancy. However, most studies have focused on the miRNAs carried by EVs, and only a limited number have determined the protein concentration of EVs.

TEM and WB observations from our study confirmed the presence of EVs in the oviduct and uterus. The characterization of these EVs was analyzed by TEM to confirm the presence and morphology of EVs, and by DLS to obtain populations of EVs of different sizes. DLS analysis showed an asymmetric size distribution of EVs, between 30 and 300 nm in diameter, which is in line with findings from other studies (Álvarez-Rodríguez et al., 2019). This distribution showed a higher frequency of larger vesicles (80%) compared to exosomes (8%) within the EVs population as in Almiñana et al. (2017). These results contradict the current measurement obtained by TEM, which differs from certain previous studies. Therefore, establishing methods to distinguish between exosomes and microvesicles is a major ongoing challenge in the field of EVs (Raposo and Stoorvogel, 2013). Besides, we believe that DLS results could be influenced by artifacts and measurements of aggregates of vesicles instead of single vesicles (Muller et al., 2014). Furthermore, some authors have shown that different techniques can give a different size distribution result and a different concentration for the same vesicle sample (van der Pol et al., 2014). Nevertheless, our results agree with previous studies that isolated exosomes and microvesicles from the bovine OF using a similar protocol to ours (centrifugating the samples at 100,000 *g* (Almiñana et al., 2017; Lopera-Vasquez et al., 2017). We also confirmed a higher proportion of larger EVs (size >150 nm) in inseminated pregnant sows compared to the non-inseminated ones, which is also in agreement with previous studies (Almiñana et al., 2017; Laezer et al., 2020). Additionally, no significant differences were observed in the size distribution of EVs in OF and UF, which is consistent with the findings of Almiñana et al. (2018). They demonstrated that the size of EVs remains constant throughout the various stages of the estrous cycle, despite the strong hormonal regulation of their molecular cargo.

### 4.1 Oviductal EVs cargo from inseminated pregnant sows

Proteomic analysis showed differences in the protein cargo of the oEVs across the sows inseminated with F1, F2 and F3. Of the 139 proteins identified in the oEVs across the three groups, 57 were exclusive in the inseminated sows, being the inseminated sows with the F1, the group with the higher number of exclusive proteins, a total of 31. Gene ontology analysis showed that the most enriched biological processes in the inseminated sows (including F1, F2 and F3) were protein stabilization, protein folding and glycolytic process. Exclusively in the oEVs from sows inseminated with F1, proteasome-mediated ubiquitin dependent protein catabolic process and positive regulation of RNA polymerase pathways were within the top five of most enriched biological processes, within these pathways we can find proteins such as 26S proteasome regulatory subunit 8 (PSMD8), proteasome 20S subunit alpha 2 (PSMA2), proteasome subunit alpha type (PSMA1), among others. The ubiquitin proteasome system (UPS) is a complex enzymatic machinery responsible for protein degradation which is involved in many biological processes such as cell cycle, cellular signalling, and transcription (Moore and Steitz, 2005). Research on UPS involvement in mammalian fertilization has demonstrated that it is related with sperm capacitation process (reviewed by Kerns et al., 2016). Moreover, UPS is also involved in the redistribution and turnover of proteins implicated in the formation of oviductal sperm reservoir, which aids in reducing polyspermy and helps in the establishment of a sequential release of freshly capacitated spermatozoa. The removal of some proteins (i.e., spermadhesins) covering the surface of the spermatozoa seems to be necessary for the disruption of the sperm-oviductal epithelium interaction, where some studies have indicated that this event is regulated by UPS-dependent proteolysis (Zigo et al., 2019; Zigo et al., 2023). Although the role of the ubiquitin proteasome catabolic process in oEVs has not been elucidated yet, this study demonstrates the presence of proteins related with such a system playing a key role in the sperm surface remodelling during capacitation (Zigo et al., 2023).

Exclusively in the oEVs from sows inseminated with the F2, the second most enriched biological process was the vesicle mediated transport pathway containing proteins previously reported (Almiñana et al., 2017) in oEVs such as Heat-shock protein family A member 8 (HSPA8), Heat-shock protein family A (HSPA70) and Syntaxin-binding protein 2 (STXBP2) which are involved in important roles in the gamete/embryo-oviduct interactions. HSPA8 belongs to a cytosolic family of chaperones that are constitutively expressed in the cytoplasm of mammalian cells under normal conditions to maintain protein structural homeostasis and are induced upon environmental stress (Nollen and Morimoto, 2002; Liman, 2017). HSPA8 protein has been found in human fallopian tubes during the menstrual cycle (Fujii et al., 2021), the bovine (Lamy et al., 2017), ovine (Soleilhavoup et al., 2016), and sow (Seytanoglu et al., 2008) cyclic oviducts. In addition, HSP70 released from porcine oviductal epithelium has been shown to enhance *in vitro* survival of boar and bull spermatozoa when spermatozoa are briefly exposed to HSP70 before interacting with oocytes (Elliott et al., 2009). These proteins also have important roles in the sperm-oviduct interaction and early embryo development (Almiñana et al., 2017). Although the mechanism of released by the oviductal cells is not known yet, authors like (Campanella et al., 2014) suggested that heat-shock proteins might be released via exosomes. These findings are consistent with the results of the present study, which identified HSPA8 and HSP70 among the relevant proteins in oEVs.

In the oEVs from pregnant sows inseminated with F3, the innate immune response pathway was exclusive of this group compared to other groups. In this pathway we found proteins which have been found in all experimental groups, such as Glyceraldehyde-3-Phosphate Dehydrogenase (GAPDH). Some researchers such as Almiñana et al. (2018) and Nakano et al. (2017) found that GAPDH mRNA is also a component of murine oEVs isolated from oviductal mesenchymal cell lines, where they demonstrated that these specific mRNAs exert a functional effect by increasing the number of ciliated cells. In addition, GAPDH has been associated with better quality embryos (Lopera-Vasquez et al., 2017), increased cryotolerance (Gutiérrez-Adán et al., 2004). Another protein found in this pathway was CD59 which is a GPI-anchored membrane protein in the cell membrane and acts as a key regulator of the complement activation cascade, thus preventing the formation of the membrane attack complex and a lytic lesion. CD59 has been found in reproductive tract cells such as sperm (Frolíková et al., 2012) and also in normal human fallopian tube, endometrium and cervical mucosa (Jensen et al., 1995). Since functional complement components are abundant in the female reproductive tract, it has been speculated that CD59 may be involved in protecting the sperm from complement-mediated damage that might be initiated by anti-sperm antibodies present in the female reproductive tract (Qian et al., 2000). Therefore, the current study demonstrated differences in protein cargo from oEVs of the sows inseminated with different sperm fractions, however essential functions involved in gamete/embryo-oviduct interactions were maintain across all the groups.

### 4.2 Uterine EVs cargo from inseminated pregnant sows

Of the 142 proteins identified in the uEVs across the three groups, 85 were exclusive in the inseminated sows, being the inseminated sows with the F1, the group with the highest number of exclusive proteins, a total of 34. In addition, 6 and 7 proteins were unique in the uEVs from sows inseminated with F2 and F3 (respectively). Gene ontology analysis showed that the most enriched biological processes (across the three groups) were protein stabilization, protein folding and cell development. These processes are related to endometrial receptivity and embryo implantation. Interestingly, GO analysis identified the protein stabilization pathway as one of the most enriched pathways in sows inseminated with F1 and F2 but not in the F3 group. Within this pathway, there are proteins such as Heat Shock Protein 90 Alpha Family Class B Member 1 (HSP90AB1), Heat Shock Protein 90 Alpha Family Class A Member 1 (HSP90AA1), Parkinson protein 7 (PARK7), Chaperonin containing TCP1, subunit 7 (CCT7), among others.

HSP90AB1 and HSP90AA1 are conserved chaperone proteins that play a key role in maintaining protein structural homeostasis and are induced by environmental stress (Neuer et al., 2000). HSP90 also plays an essential role in the controlled inflammatory response required for conceptus implantation and trophoblast growth (Jee et al., 2021). For example, HSPAA901 is a co-factor for steroid hormone receptors and is released from the receptor complex upon ligand hormone-receptor interaction produced by uterine cells (Neuer et al., 2000). This protein has previously been identified in the porcine OF (Mondéjar et al., 2013), and it has been suggested to be released via exosomes (Campanella et al., 2012) or lipid rafts (Pralle et al., 2000). On the other hand, Chaperonin containing TCP1 is necessary for folding newly synthesized proteins, including actin and tubulin (Yam et al., 2008). This has also been previously identified in the porcine endometrium of pregnant sows (Pierzchała et al., 2021), being higher expressed in healthy pregnancy compared to pregnancy lost. Therefore, these proteins seem to be involved in embryo maternal interaction and the signaling of maternal recognition of pregnancy.

In the uEVs from pregnant sows inseminated with F1, the protein localisation to plasma membrane pathway was only found in this group. Rap1, as a member of the GTPase family, is normally in the active GTP-bound form, which is the main regulator of cell-cell junction (Gaonac’h-Lovejoy et al., 2020), and cell adhesion (Boettner and Van Aelst, 2009). Moreover, Rap1 has been related to embryo implantation, as Rap1 can induce cell–cell junction stabilization (Gaonac’h-Lovejoy et al., 2020). The activation of Rap1 has been shown to be involved in the establishment of cell polarity (Freeman et al., 2017), where this epithelial cell polarity is rebuilt during uterine repair, then suggesting that Rap1a is involved in the reparation of endometrial injury (Zhang et al., 2023) including physiological processes occurring in the establishment of pregnancy such as embryo implantation and the development of uterine fibroids. Moreover, some pathways related to actin filament organization were found in the sows inseminated with F2 and F3. This suggests that all the fractions of the ejaculate are mediating in the uterine remodulation of the tissue during the establishment of pregnancy.

In summary, the results of this work demonstrated that the porcine EVs isolated from OF and UF exhibit different protein cargo depending on the semen plasma in the AI dose. We were also able to demonstrate that the AI with different sperm fractions results in the secretion of EVs with specific protein content, and these proteins are closely associated with reproductive processes.

## 5 Conclusion

In conclusion, to the best of our knowledge, the present study provides novel insights into the differential expression of some EVs-induced proteins in pregnant sows inseminated with three different accumulative fractions of the boar ejaculate compared to non-pregnant sows. These results contribute to a better understanding of the regulation of oviduct and uterine physiology, elucidating candidate proteins that may interact with gametes and embryos, thereby modulating reproductive events involved in fertilization. Our findings include already-known fertility-related proteins, several new candidates that may modulate sperm survival and embryo development, and functions related to fertilization. Further studies will be required to determine the exact role of these proteins and their interaction mechanism by binding or fusion with the sperm, the oocyte, or even the female genital tract epithelium. Understanding the contribution of EVs to the fine-tuning of the oviductal and uterine environment may help to better mimic the *in vitro* environment during *in vitro* production of porcine embryos to enhance their quality.

## Data Availability

The datasets presented in this study can be found in online repositories. The names of the repository/repositories and accession number(s) can be found below: https://www.ebi.ac.uk/pride/archive/, PXD044639.
