# Supplementary material for: Different seminal ejaculated fractions in artificial insemination condition the protein cargo of oviductal and uterine extracellular vesicles in pig
Source: Front Cell Dev Biol. 2023 Oct 6;11:1231755. doi: 10.3389/fcell.2023.1231755 (PMC10587466; doi:10.3389/fcell.2023.1231755)
Supplement: Supplementary file 1 [file Presentation1.zip › Supplementary File 1.DOCX]

Supplementary Material

Different sperm ejaculated fractions in artificial insemination condition the protein cargo of oviductal and uterine extracellular vesicles in pig

S. Toledo^1^, C. Luongo^1^, L. Abril-Parreño^1^, C. Soriano-Úbeda^2*^ and C. Matás^1,3*^

*** Correspondence:** Corresponding Author: C. Soriano-Úbeda ([c.soriano.ubeda@unileon.es](mailto:c.soriano.ubeda@unileon.es)); C. Matás ([cmatas@um.es](mailto:cmatas@um.es))

**Supplementary table 1.** Exclusive and common proteins identified in extracellular vesicles from oviductal fluid (oEVs) in sows (n=20) artificially inseminated (AI-sows) with different seminal doses from boars (n = 6). Non-inseminated sows were considered as control (C-sows).

| **Experimental group** | **Protein name** | **Accession number** |
| --- | --- | --- |
| **C-sows** | 14-3-3 protein theta | A0A4X1UDE4 |
|  | 26S proteasome AAA-ATPase subunit RPT1 | A0A287BR45 |
|  | 26S proteasome non-ATPase regulatory subunit 1 | A0A0B8RTB9 |
|  | 26S proteasome regulatory subunit 8 | A0A287AVG1 |
|  | 40S ribosomal protein S11 | A0A4X1VTS2 |
|  | 40S ribosomal protein S13 | A0A480V540 |
|  | 40S ribosomal protein S14 | A0A287BHE7 |
|  | 40S ribosomal protein S16 | A0A4X1SXI6 |
|  | 40S ribosomal protein S17 | A0A4X1ULB7 |
|  | 40S ribosomal protein S18 | P62272 |
|  | 40S ribosomal protein S19 | A0A286ZX70 |
|  | 40S ribosomal protein S23 | A0A4X1T9H7 |
|  | 40S ribosomal protein S3 | A0A4X1UN85 |
|  | 40S ribosomal protein S3a | A0A287B4K6 |
|  | 40S ribosomal protein S4 | A0A287B771 |
|  | 40S ribosomal protein S5 | A0A287AEM2 |
|  | 40S ribosomal protein S6 | F2Z5Q6 |
|  | 40S ribosomal protein S8 | A0A4X1W9D1 |
|  | 40S ribosomal protein S9 | A0A287BPF2 |
|  | 60S acidic ribosomal protein P0 | A0A287AY54 |
|  | 60S acidic ribosomal protein P1 | A0A4X1T568 |
|  | 60S acidic ribosomal protein P2 | A0A287B7U0 |
|  | 60S ribosomal protein L11 | A0A4X1VYM5 |
|  | 60S ribosomal protein L12 | A0A0B8RZ72 |
|  | 60S ribosomal protein L14 | A0A286ZW72 |
|  | 60S ribosomal protein L17 | A0A287B386 |
|  | 60S ribosomal protein L18 | A0A4X1VZE3 |
|  | 60S ribosomal protein L18a | A0A287APR1 |
|  | 60S ribosomal protein L23 | A0A4X1ULC0 |
|  | 60S ribosomal protein L29 | A0A287ANI9 |
|  | 60S ribosomal protein L30 | A0A287B6Y1 |
|  | 60S ribosomal protein L31 | A0A287A7F5 |
|  | 60S ribosomal protein L5 | A0A287ASD0 |
|  | 60S ribosomal protein L6 | A0A287BBI3 |
|  | 60S ribosomal protein L9 | A0A4X1V3Z6 |
|  | 78 kDa glucose-regulated protein | A0A287BIL8 |
|  | AAA domain-containing protein | A0A286ZT52 |
|  | AHNAK nucleoprotein | A0A287A608 |
|  | Alpha-1,4 glucan phosphorylase | A0A287AL94 |
|  | Alpha-2-macroglobulin isoform X1 | A0A480KMH7 |
|  | AP-2 complex subunit alpha | A0A4X1VSA7 |
|  | Arginine--tRNA ligase, cytoplasmic | A0A480TM87 |
|  | Armadillo repeat containing 4 | A0A287BDJ3 |
|  | Aspartate carbamoyltransferase | A0A480EZK3 |
|  | Aspartate--tRNA ligase, cytoplasmic | A0A286ZYS2 |
|  | Brain acid soluble protein 1 | A0A287ALA0 |
|  | BRO1 domain-containing protein | A0A287A5B4 |
|  | Cadherin-13 | A0A287ALP4 |
|  | Choline transporter-like protein 4 | A0A2C9F351 |
|  | Coatomer subunit alpha | A0A480EME6 |
|  | Copine 3 | A0A286ZNB9 |
|  | DnaJ heat shock protein family (Hsp40) member A1 | A0A4X1UQS4 |
|  | DnaJ heat shock protein family (Hsp40) member A2 | A0A4X1TXP8 |
|  | DnaJ homolog subfamily A member 4 isoform 1 | A0A480ZY99 |
|  | Dolichyl-diphosphooligosaccharide--protein glycosyltransferase subunit 2 | A0A480SN74 |
|  | E3 ubiquitin/ISG15 ligase TRIM25 | A0A4X1SGH3 |
|  | G protein subunit alpha 13 | A0A287A853 |
|  | G protein subunit alpha i3 | A0A4X1TDW5 |
|  | Galectin | A0A4X1WD32 |
|  | Galectin-3-binding protein | A0A287A604 |
|  | Glutamyl-tRNA synthetase | A0A286ZQW0 |
|  | Guanine nucleotide binding protein (G protein), beta polypeptide 1 | A0A0B8RZJ8 |
|  | H1.4 linker histone, cluster member | A0A286ZZB7 |
|  | Histone H2A | A0A4X1SJ18 |
|  | Histone H4 | A0A480TD90 |
|  | IF rod domain-containing protein | A0A287BF12 |
|  | IgG heavy chain | L8B180 |
|  | Ig-like domain-containing protein | A0A075B7H9 |
|  | Immunoglobulin heavy constant mu | A0A0A0MY58 |
|  | Isoleucyl-tRNA synthetase | A0A287A4W5 |
|  | Isoprenylcysteine carboxyl methyltransferase | A0A5G2QFP8 |
|  | Joining chain of multimeric IgA and IgM | A0A287B5V2 |
|  | Keratin, type I cytoskeletal 19 | A0A480IFL7 |
|  | Kinetochore associated 1 | A0A287AML8 |
|  | Neuroblast differentiation-associated protein AHNAK isoform X1 | A0A480Z467 |
|  | Poly(rC) binding protein 1 | A0A4X1W9X7 |
|  | Ras homolog family member A | A0A4X1ST20 |
|  | Ribosomal protein | A0A287BSA8 |
|  | Ribosomal protein L23a | A0A286ZL51 |
|  | Ribosomal protein L7 | A0A287A8T0 |
|  | Ubiquitin C | A0A0B8RT13 |
| **AI-sows** | 14-3-3 protein zeta/delta | A0A480PLY3 |
|  | 6-phosphogluconate dehydrogenase, decarboxylating | A0A480X5G4 |
|  | Actin beta (Fragments) | Q7M3B0 |
|  | Actin, gamma 1 | A0A0B8RTA2 |
|  | Adenylyl cyclase-associated protein | A0A0B8S0B1 |
|  | Alcohol dehydrogenase [NADP(+)] | A0A287A3Y9 |
|  | Brain-specific angiogenesis inhibitor 1-associated protein 2-like protein 1 | A0A480WV84 |
|  | Calcium-activated neutral proteinase 2 | A0A480JWG2 |
|  | Carbonyl reductase 3 | A0A0K1TQQ7 |
|  | CCT-alpha | A0A165DE67 |
|  | CRIP2 | A0A165DE67 |
|  | Dimethylargininase | A0A0D5CDB2 |
|  | Fibrinogen beta chain | A0A480TMT6 |
|  | Fructose-bisphosphate aldolase | A0A286ZWI1 |
|  | Gc-globulin | A0A287AHK1 |
|  | GTP-binding nuclear protein Ran | A0A4X1UF70 |
|  | Heat shock 110 kDa protein | A0A286ZMW4 |
|  | Heat shock 70 kDa protein 1B | A0A480ZTG9 |
|  | Hemoglobin subunit epsilon | P02101 |
|  | Inositol-3-phosphate synthase 1 | A0A0B8RVT0 |
|  | Inter-alpha-trypsin inhibitor heavy chain H2 | A0A480R9R0 |
|  | Keratin 15 | A0A286ZYN0 |
|  | Keratin 79 | A0A287A2X0 |
|  | Leucine zipper transcription factor-like protein 1 | A0A480UCV4 |
|  | L-lactate dehydrogenase | A0A4X1VAI4 |
|  | LPS-responsive vesicle trafficking, beach and anchor containing | A0A0B8RTI4 |
|  | Mucin 4, cell surface associated | A0A287B5M2 |
|  | Myosin regulatory light chain 12B | A0A480LQL7 |
|  | Myosin-9 | A0A480IS92 |
|  | Na(+)-dependent phosphate cotransporter 2B | A0A4X1V982 |
|  | NIMA related kinase 1 | A0A286ZYY1 |
|  | Peptidyl-prolyl cis-trans isomerase E | A0A286ZJY9 |
|  | Peroxiredoxin-2 | A0A287A690 |
|  | Peroxiredoxin-5 | A0A4X1V8R7 |
|  | Phosphoglycerate mutase | A0A287AJQ2 |
|  | Podocalyxin | F1SNF3 |
|  | Polo like kinase 4 | A0A286ZIK8 |
|  | Profilin | A0A4X1UWL1 |
|  | Rab GDP dissociation inhibitor | A0A287B4B7 |
|  | Rho GDP dissociation inhibitor alpha | A0A287AQC2 |
|  | Serine/threonine-protein phosphatase | A0A1B2TT50 |
|  | Serine/threonine-protein phosphatase 2A 65 kDa regulatory subunit A alpha isoform | A0A287BB72 |
|  | S-formylglutathione hydrolase | A0A4X1SH01 |
|  | Solute carrier family 15 member 2 | A0A287BMQ3 |
|  | Stomatin | A0A287AE47 |
|  | Syndecan binding protein | A0A286ZKH9 |
|  | Syntaxin-binding protein 2 | A0A287BB33 |
|  | T-complex protein 1 subunit eta | A0A480KDY2 |
|  | T-complex protein 1 subunit zeta | A0A480MNA5 |
|  | Tr-type G domain-containing protein | A0A286ZUI3 |
|  | Tubulin alpha-1A chain | P02550 |
|  | Tyrosine 3-monooxygenase/tryptophan 5-monooxygenase activation protein zeta | A0A287A0Q6 |
|  | Uncharacterized protein | A0A286ZJL9 |
|  | Uncharacterized protein | A0A4X1VKL9 |
|  | Uncharacterized protein | A0A287BQU9 |
|  | UTP-glucose-1-phosphate uridylyltransferase | A0A286ZI08 |
|  | Zinc finger MYND-type containing 10 | A0A286ZWL4 |
| **C- and AI-sows** | 14_3_3 domain-containing protein | A0A287A2R9 |
|  | 15S Mg(2+)-ATPase p97 subunit | A0A286ZSB5 |
|  | 26S proteasome AAA-ATPase subunit RPT3 | A0A287B4P8 |
|  | 2-phospho-D-glycerate hydro-lyase | A0A0B8RSY9 |
|  | 3-hydroxyacyl-[acyl-carrier-protein] dehydratase | A0A480M011 |
|  | 40S ribosomal protein S2 | A0A287ASU4 |
|  | 40S ribosomal protein S7 | A0A287A9Y6 |
|  | 60S ribosomal protein L4 | A0A0B8RT95 |
|  | 60S ribosomal protein L7a | A0A0B8RSA9 |
|  | Actin gamma 2, smooth muscle | A0A286ZWJ1 |
|  | Actin-depolymerizing factor | A0A286ZIC1 |
|  | ADP ribosylation factor 1 | A0A286ZTI3 |
|  | Albumin | A0A286ZT13 |
|  | Aldehyde dehydrogenase | A0A0K1TQQ2 |
|  | Aldehyde dehydrogenase 1 family member A1 | A0A287BRG6 |
|  | Aminopeptidase | A0A0B8RZR8 |
|  | Angiotensin-converting enzyme | A0A480IQM9 |
|  | Annexin | A0A286ZJV6 |
|  | ATP-dependent 6-phosphofructokinase | A0A286ZZL7 |
|  | Calcium-transporting ATPase | A0A481AYZ5 |
|  | Calcyphosin isoform a | A0A287AHT9 |
|  | Calmodulin 3 | A0A287AHS0 |
|  | CD59 glycoprotein | O62680 |
|  | Cell division control protein 42 homolog | Q007T2 |
|  | Chloride intracellular channel protein | A0A0B8S0A2 |
|  | Clathrin heavy chain | A0A4X1SRE7 |
|  | Clusterin | A0A4X1TMH0 |
|  | Cofilin, non-muscle isoform | A0A286ZVE7 |
|  | Creatine kinase | A0A4X1TS58 |
|  | Cytokeratin-1 | A5A758 |
|  | D-3-phosphoglycerate dehydrogenase | A0A480HK41 |
|  | Deleted in malignant brain tumors 1 protein | A0A480JBS1 |
|  | Dynein cytoplasmic 1 heavy chain 1 | A0A287B9W3 |
|  | Elongation factor 2 | A0A287AWI9 |
|  | Ezrin | A0A480UUL9 |
|  | Fibrinogen alpha chain | A0A4X1SSQ4 |
|  | Fibrinogen C-terminal domain-containing protein | A0A4X1SSS2 |
|  | G protein subunit alpha i2 | A0A287B697 |
|  | G protein-coupled receptor class C group 5 member C | A0A286ZX63 |
|  | GLOBIN domain-containing protein | A0A4X1SXN8 |
|  | Glyceraldehyde-3-phosphate dehydrogenase | A0A076KRX0 |
|  | Glycoprotein M6A | A0A4X1VSA1 |
|  | Guanine nucleotide binding protein (G protein), q polypeptide | A0A0B8RZJ8 |
|  | Guanine nucleotide-binding protein G(I)/G(S)/G(T) subunit beta-2 | A0A0B8RZJ8 |
|  | HATPase_c domain-containing protein | A0A286ZKC5 |
|  | Heat shock 27 kDa protein | A0A2C9F366 |
|  | Heat shock protein family A (Hsp 70) member 8 | A0A286ZPC8 |
|  | Heat shock protein HSP 90-alpha | A0A287AQK7 |
|  | Hemoglobin subunit alpha | P01965 |
|  | IQ motif containing GTPase activating protein 1 | A0A4X1UF40 |
|  | Keratin 8 | A0A287A8E9 |
|  | Ketimine reductase mu-crystallin | A0A480NX20 |
|  | Kinesin light chain 2 | A0A286ZJ53 |
|  | L-lactate dehydrogenase B chain | A0A287AQS1 |
|  | Major vault protein | A0A480Z666 |
|  | Malate dehydrogenase | A0A480JV95 |
|  | Myosin light polypeptide 6 isoform 2 | A0A480I0Y2 |
|  | Na(+)/H(+) exchange regulatory cofactor NHE-RF | A0A287AAH4 |
|  | Oviduct-specific glycoprotein | A0A286ZZ91 |
|  | Peptidyl-prolyl cis-trans isomerase | A0A4X1UCS5 |
|  | Potassium channel tetramerization domain containing 12 | F1RHE6 |
|  | Proteasome subunit alpha type | A0A5G2QI19 |
|  | Proteasome 20S subunit alpha 2 | A0A4X1UJP8 |
|  | Protein GNAS isoform GNASS | A0A480UPA5 |
|  | Protein S100 | A0A5G2QQA2 |
|  | Protein-arginine deiminase | A0A287B865 |
|  | Pyruvate kinase | A0A0B8S031 |
|  | RuvB-like helicase | A0A287B8A9 |
|  | Small integral membrane protein 22 | A0A4X1VTF1 |
|  | Sodium/nucleoside cotransporter | A0A4X1UYE9 |
|  | Thioredoxin domain-containing protein | A0A4X1VDC5 |
|  | Transket_pyr domain-containing protein | A0A4X1VH46 |
|  | Triosephosphate isomerase | A0A286ZRV2 |
|  | Trypsinogen isoform X1 | A0A287B5W2 |
|  | Tubulin alpha chain | A0A4X1W776 |
|  | Tubulin beta chain | A0A287A275 |
|  | Tubulin polymerization-promoting protein family member 3 | A0A4X1UK58 |
|  | Tyrosine 3-monooxygenase/tryptophan 5-monooxygenase activation protein, beta | A0A0B8RVD2 |
|  | Uncharacterized protein | A0A4X1U5T5 |
|  | Uncharacterized protein | A0A287ALC1 |
|  | Uncharacterized protein | A0A4X1T940 |
|  | Uncharacterized protein | A0A4X1TWK8 |

**Supplementary table 2.** Exclusive and common proteins identified in extracellular vesicles from oviductal fluid (oEVs) in sows (n=20) inseminated with different semen doses from boars (n = 6) according to the accumulative seminal fraction(s) used for artificial insemination (AI): F1, composed by the sperm-rich fraction (SRF); F2, composed by F1 plus the intermediate fraction; F3, composed by F2 plus the post-SRF.

| **Experimental group** | **Protein name** | **Accession number** |
| --- | --- | --- |
| **AI-F1** | 14-3-3 protein zeta/delta | A0A480PLY3 |
|  | 26S proteasome AAA-ATPase subunit RPT3 | A0A287B4P8 |
|  | 6-phosphogluconate dehydrogenase, decarboxylating | A0A480X5G4 |
|  | Actin beta (Fragments) | Q7M3B0 |
|  | ATP-dependent 6-phosphofructokinase | A0A286ZZL7 |
|  | Brain-specific angiogenesis inhibitor 1-associated protein 2-like protein 1 | A0A480WV84 |
|  | Carbonyl reductase 3 | A0A0K1TQQ7 |
|  | CCT-alpha | A0A165DE67 |
|  | Cofilin, non-muscle isoform | A0A286ZVE7 |
|  | CRIP2 | A0A165DE67 |
|  | Cytokeratin-1 | A5A758 |
|  | D-3-phosphoglycerate dehydrogenase | A0A480HK41 |
|  | Hemoglobin subunit alpha | P01965 |
|  | Inter-alpha-trypsin inhibitor heavy chain H2 | A0A480R9R0 |
|  | Keratin 15 | A0A286ZYN0 |
|  | Keratin 79 | A0A287A2X0 |
|  | Leucine zipper transcription factor-like protein 1 | A0A480UCV4 |
|  | L-lactate dehydrogenase | A0A4X1VAI4 |
|  | LPS-responsive vesicle trafficking, beach and anchor containing | A0A0B8RTI4 |
|  | Peptidyl-prolyl cis-trans isomerase E | A0A286ZJY9 |
|  | Polo like kinase 4 | A0A286ZIK8 |
|  | Proteasome subunit alpha type | A0A5G2QI19 |
|  | Serine/threonine-protein phosphatase | A0A1B2TT50 |
|  | S-formylglutathione hydrolase | A0A4X1SH01 |
|  | T-complex protein 1 subunit eta | A0A480KDY2 |
|  | T-complex protein 1 subunit zeta | A0A480MNA5 |
|  | Tubulin alpha-1A chain | P02550 |
|  | Zinc finger MYND-type containing 10 | A0A286ZWL4 |
|  | Uncharacterized protein | A0A286ZJL9 |
|  | Uncharacterized protein | A0A4X1T940 |
|  | Uncharacterized protein | A0A4X1VKL9 |
| **AI-F2** | Actin gamma 2, smooth muscle | A0A286ZWJ1 |
|  | GTP-binding nuclear protein Ran | A0A4X1UF70 |
|  | Keratin 8 | A0A287A8E9 |
|  | Kinesin light chain 2 | A0A286ZJ53 |
|  | NIMA related kinase 1 | A0A286ZYY1 |
|  | Peroxiredoxin-2 | A0A287A690 |
|  | Rab GDP dissociation inhibitor | A0A287B4B7 |
|  | Rho GDP dissociation inhibitor alpha | A0A287AQC2 |
|  | Syndecan binding protein | A0A287BQU9 |
| **AI-F3** | 2-phospho-D-glycerate hydro-lyase | A0A0B8RSY9 |
|  | 40S ribosomal protein S2 | A0A287ASU4 |
|  | 40S ribosomal protein S7 | A0A287A9Y6 |
|  | 60S ribosomal protein L4 | A0A0B8RT95 |
|  | 60S ribosomal protein L7a | A0A0B8RSA9 |
|  | Aldehyde dehydrogenase | A0A0K1TQQ2 |
|  | Fibrinogen alpha chain | A0A4X1SSQ4 |
|  | Fibrinogen C-terminal domain-containing protein | A0A4X1SSS2 |
|  | Glycoprotein M6A | A0A4X1VSA1 |
|  | Heat shock 70 kDa protein 1B | A0A480ZTG9 |
|  | Hemoglobin subunit epsilon | A5GFW1 |
|  | Major vault protein | A0A480Z666 |
|  | Peptidyl-prolyl cis-trans isomerase | A0A4X1UCS5 |
|  | Podocalyxin | F1SNF3 |
|  | Profilin | A0A4X1UWL1 |
|  | Protein S100 | A0A5G2QQA2 |
|  | Small integral membrane protein 22 | A0A4X1VTF1 |
|  | Solute carrier family 15 member 2 | A0A287BMQ3 |
|  | Stomatin | A0A287AE47 |
|  | Tubulin polymerization-promoting protein family member 3 | A0A4X1UK58 |
|  | Tyrosine 3-monooxygenase/tryptophan 5-monooxygenase activation protein zeta | A0A287A0Q6 |
|  | Tyrosine 3-monooxygenase/tryptophan 5-monooxygenase activation protein, beta | A0A0B8RVD2 |
|  | UTP--glucose-1-phosphate uridylyltransferase | A0A286ZI08 |
|  | Uncharacterized protein | A0A4X1TWK8 |
| **AI-F1, AI-F2, and AI-F3** | 14_3_3 domain-containing protein | A0A287A2R9 |
|  | 15S Mg(2+)-ATPase p97 subunit | A0A286ZSB5 |
|  | 3-hydroxyacyl-[acyl-carrier-protein] dehydratase | A0A480M011 |
|  | Actin, gamma 1 | A0A0B8RTA2 |
|  | Albumin | A0A286ZT13 |
|  | Aldehyde dehydrogenase 1 family member A1 | A0A287BRG6 |
|  | Aminopeptidase | A0A0B8RZR8 |
|  | Annexin | A0A286ZJV6 |
|  | Calcium-activated neutral proteinase 2 | A0A480JWG2 |
|  | Calcium-transporting ATPase | A0A481AYZ5 |
|  | Calcyphosin isoform a | A0A287AHT9 |
|  | Calmodulin 3 | A0A287AHS0 |
|  | Chloride intracellular channel protein | A0A0B8S0A2 |
|  | Clathrin heavy chain | A0A4X1SRE7 |
|  | Clusterin | A0A4X1TMH0 |
|  | Dimethylargininase | A0A0D5CDB2 |
|  | Elongation factor 2 | A0A287AWI9 |
|  | G protein-coupled receptor class C group 5 member C | A0A286ZX63 |
|  | GLOBIN domain-containing protein | A0A4X1SXN8 |
|  | Glyceraldehyde-3-phosphate dehydrogenase | A0A076KRX0 |
|  | Guanine nucleotide binding protein (G protein), q polypeptide | A0A0B8S079 |
|  | HATPase_c domain-containing protein | A0A286ZKC5 |
|  | Heat shock protein family A (Hsp 70) member 8 | A0A286ZPC8 |
|  | Heat shock protein HSP 90-alpha | A0A287AQK7 |
|  | Inositol-3-phosphate synthase 1 | A0A0B8RVT0 |
|  | Ketimine reductase mu-crystallin | A0A480NX20 |
|  | Malate dehydrogenase | A0A480JV95 |
|  | Myosin-9 | A0A480IS92 |
|  | Na(+)/H(+) exchange regulatory cofactor NHE-RF | A0A287AAH4 |
|  | Oviduct-specific glycoprotein | A0A286ZZ91 |
|  | Peroxiredoxin-5 | A0A4X1V8R7 |
|  | Protein GNAS isoform GNASS | A0A480UPA5 |
|  | Protein-arginine deiminase | A0A287B865 |
|  | Pyruvate kinase | A0A0B8S031 |
|  | Serine/threonine-protein phosphatase 2A 65 kDa regulatory subunit A alpha isoform | A0A287BB72 |
|  | Sodium/nucleoside cotransporter | A0A4X1UYE9 |
|  | Thioredoxin domain-containing protein | A0A4X1VDC5 |
|  | Transket_pyr domain-containing protein | A0A4X1VH46 |
|  | Triosephosphate isomerase | A0A286ZRV2 |
|  | Tr-type G domain-containing protein | A0A286ZUI3 |
|  | Trypsinogen isoform X1 | A0A287B5W2 |
|  | Tubulin alpha chain | A0A4X1W776 |
|  | Tubulin beta chain | A0A287A275 |
|  | Uncharacterized protein | A0A4X1U5T5 |
| **AI-F1 and AI-F2** | Alcohol dehydrogenase [NADP(+)] | A0A287A3Y9 |
|  | Deleted in malignant brain tumors 1 protein | A0A480JBS1 |
|  | Phosphoglycerate mutase | A0A287AJQ2 |
|  | Proteasome 20S subunit alpha 2 | A0A4X1UJP8 |
|  | RuvB-like helicase | A0A287B8A9 |
| **AI-F1 and AI-F3** | Actin-depolymerizing factor | A0A286ZIC1 |
|  | Adenylyl cyclase-associated protein | A0A0B8S0B1 |
|  | CD59 glycoprotein | O62680 |
|  | Creatine kinase | A0A4X1TS58 |
|  | Fibrinogen beta chain | A0A480TMT6 |
|  | Fructose-bisphosphate aldolase | A0A286ZWI1 |
|  | Gc-globulin | A0A287AHK1 |
|  | Guanine nucleotide-binding protein G(I)/G(S)/G(T) subunit beta-2 | A0A480ZG01 |
|  | Heat shock 110 kDa protein | A0A286ZMW4 |
|  | Mucin 4, cell surface associated | A0A287B5M2 |
|  | Myosin light polypeptide 6 isoform 2 | A0A480I0Y2 |
|  | Uncharacterized protein | A0A287ALC1 |
| **AI-F2 and AI-F3** | ADP ribosylation factor 1 | A0A286ZTI3 |
|  | Angiotensin-converting enzyme | A0A480IQM9 |
|  | Cell division control protein 42 homolog | Q007T2 |
|  | Dynein cytoplasmic 1 heavy chain 1 | A0A287B9W3 |
|  | Ezrin | A0A480UUL9 |
|  | G protein subunit alpha i2 | A0A287B697 |
|  | Heat shock 27 kDa protein | A0A2C9F366 |
|  | IQ motif containing GTPase activating protein 1 | A0A4X1UF40 |
|  | L-lactate dehydrogenase B chain | A0A287AQS1 |
|  | Myosin regulatory light chain 12B | A0A480LQL7 |
|  | Na(+)-dependent phosphate cotransporter 2B | A0A4X1V982 |
|  | Potassium channel tetramerization domain containing 12 | F1RHE6 |
|  | Syntaxin-binding protein 2 | A0A287BB33 |

**Supplementary table 3.** Exclusive and common proteins identified in extracellular vesicles from uterine fluid (uEVs) in sows (n=20) artificially inseminated (AI-sows) with different seminal doses from boars (n = 6). Non-inseminated sows were considered as control (C-sows).

| **Experimental group** | | **Protein name** | | **Accession number** | |
| --- | --- | --- | --- | --- | --- |
| **C-sows** | | 60S acidic ribosomal protein P1 | | A0A4X1T568 | |
|  |  | Adipsin | | A0A480UQ82 | |
|  |  | Beta-2-microglobulin | | A0A287B7B9 | |
|  |  | BPTI/Kunitz inhibitor domain-containing protein | | A0A4X1TWT4 | |
|  |  | Carboxypeptidase M | | A0A287APR3 | |
|  |  | Ceruloplasmin | | A0A481CII4 | |
|  |  | Clathrin heavy chain | | A0A4X1SRE7 | |
|  |  | Clusterin | | A0A4X1V775 | |
|  |  | Contactin 3 | | A0A287BDF0 | |
|  |  | E3 ubiquitin-protein ligase UBR4 | | A0A480IRP8 | |
|  |  | Fibrocystin-L | | A0A4X1TCE8 | |
|  |  | Fibronectin | | A0A480HYV8 | |
|  |  | Galectin-3-binding protein | | A0A287A604 | |
|  |  | Glucosaminyl (N-acetyl) transferase 2 (I blood group) | | A0A287A5Z0 | |
|  |  | GTP-binding nuclear protein Ran | | A0A4X1UF70 | |
|  |  | Heat shock protein family A (Hsp70) member 8 | | A0A286ZPC8 | |
|  |  | IgA constant region | | A0A287B626 | |
|  |  | Ig-like domain-containing protein | | A0A286ZK41 | |
|  |  | Immunoglobulin heavy constant mu | | A0A0A0MY58 | |
|  |  | Joining chain of multimeric IgA and IgM | | A0A287B5V2 | |
|  |  | L-lactate dehydrogenase B chain | | A0A287AQS1 | |
|  |  | MARVEL domain-containing protein | | A0A286ZMT0 | |
|  |  | Metalloendopeptidase | | A0A286ZZE3 | |
|  |  | N-acetylated alpha-linked acidic dipeptidase 2 | | A0A286ZMF8 | |
|  |  | Nectin cell adhesion molecule 2 | | A0A4X1W1F9 | |
|  |  | Polymeric immunoglobulin receptor | | A0A0E3M2Q4 | |
|  |  | Serine protease 8 | | A0A286ZNI7 | |
|  |  | T-complex protein 1 subunit gamma | | A0A287AMZ2 | |
|  |  | Trypsinogen isoform X1 | | A0A287B5W2 | |
|  |  | Vitronectin | | A0A480QP96 | |
| **AI-sows** | | 14_3_3 domain-containing protein | | A0A4X1UM41 | |
|  |  | 14-3-3 protein zeta/delta | | A0A480PLY3 | |
|  |  | 2',3'-cyclic-nucleotide 3'-phosphodiesterase | | A0A286ZZ12 | |
|  |  | 2-phospho-D-glycerate hydro-lyase | | A0A0B8RSY9 | |
|  |  | 3-hydroxyacyl-[acyl-carrier-protein] dehydratase | | A0A480M011 | |
|  |  | 40S ribosomal protein S8 | | A0A4X1SUR2 | |
|  |  | 60S acidic ribosomal protein P2 | | A0A287B7U0 | |
|  |  | Actin-depolymerizing factor | | A0A286ZIC1 | |
|  |  | Actin-like protein 3 | | A0A0B8RZS4 | |
|  |  | Adenylyl cyclase-associated protein | | A0A0B8S0B1 | |
|  |  | Adseverin | | A0A287A900 | |
|  |  | Ammonium transporter Rh type C | | A0A4X1U0V5 | |
|  |  | ATP-binding cassette sub-family G member 2 | | A0A4X1U7U2 | |
|  |  | Brain-specific angiogenesis inhibitor 1-associated protein 2 | | A0A287BKX8 | |
|  |  | Calcium-activated neutral proteinase 1 | | A0A480EV23 | |
|  |  | Calmodulin 3 | | A0A287AHS0 | |
|  |  | cAMP-dependent protein kinase type II-alpha regulatory subunit | | A0A4X1T0V7 | |
|  |  | CCT-alpha | | A0A287AJY2 | |
|  |  | Charged multivesicular body protein 6 | | A0A4X1UDW0 | |
|  |  | Chloride intracellular channel protein | | A0A0B8S0A2 | |
|  |  | Cofilin, non-muscle isoform | | A0A286ZVE7 | |
|  |  | Cystathionine beta-synthase | | A0A287AQV4 | |
|  |  | Cytosolic non-specific dipeptidase isoform X1 | | A0A480SKP2 | |
|  |  | DnaJ heat shock protein family (Hsp40) member A1 | | A0A4X1UQS4 | |
|  |  | DNAJ heat shock protein family (Hsp40) member A2 | | A0A4X1TXQ3 | |
|  |  | Elongation factor 2 | | A0A287AWI9 | |
|  |  | Epidermal growth factor receptor pathway substrate 8 | | A0A287BJX5 | |
|  |  | EPS8 like 2 | | A0A286ZVR9 | |
|  |  | Ezrin | | A0A480UUL9 | |
|  |  | Filamin B | | A0A286ZPG4 | |
|  |  | G protein subunit alpha i3 | | A0A4X1TDW5 | |
|  |  | Glia maturation factor | | A0A287A6Y3 | |
|  |  | Glucose-6-phosphate isomerase | | A0A480S836 | |
|  |  | Glutaredoxin | | F2XWZ9 | |
|  |  | Guanine nucleotide binding protein (G protein), beta polypeptide 1 | | A0A0B8RZJ8 | |
|  |  | HATPase_c domain-containing protein | | A0A286ZKC5 | |
|  |  | Heat shock protein family A (Hsp70) member 8 | | A0A286ZPC8 | |
|  |  | Heat shock protein HSP 90-alpha | | A0A287AQK7 | |
|  |  | Heterogeneous nuclear ribonucleoprotein K | | A0A4X1UTE2 | |
|  |  | Integrin-associated protein | | A0A286ZQ17 | |
|  |  | Interferon-induced transmembrane protein 1 | | A0A142EGG5 | |
|  |  | Maillard deglycase | | A0A4X1W8H4 | |
|  |  | MARCKS like 1 | | A0A287B4N8 | |
|  |  | Mitogen-activated protein kinase | | A0A286ZPF5 | |
|  |  | Mucin 4, cell surface associated | | A0A287B5M2 | |
|  |  | Myosin-10 | | A0A480HB69 | |
|  |  | Myristoylated alanine rich protein kinase C substrate | | A0A287BRL8 | |
|  |  | Na(+)/H(+) exchange regulatory cofactor NHE-RF | | A0A287AAH4 | |
|  |  | Na(+)-dependent phosphate cotransporter 2B | | A0A4X1V982 | |
|  |  | Neuroblast differentiation-associated protein AHNAK isoform X1 | | A0A480Z467 | |
|  |  | Niban-like protein 1 | | A0A0B8RSY4 | |
|  |  | Non-specific serine/threonine protein kinase | | A0A287APG0 | |
|  |  | PDZ domain containing 1 | | A0A287BAJ3 | |
|  |  | Pendrin | | A0A480E679 | |
|  |  | Peroxiredoxin-1 | | A0A286ZND5 | |
|  |  | Phosphoglycerate mutase | | A0A287AJQ2 | |
|  |  | Phosphoserine aminotransferase | | A0A480S1F9 | |
|  |  | PKS_ER domain-containing protein | | A0A4X1TWX8 | |
|  |  | Plastin 1 | | A0A287APD4 | |
|  |  | Proline rich transmembrane protein 1B | | A0A287ADX3 | |
|  |  | Protein GNAS isoform GNASS | | A0A480UPA5 | |
|  |  | Protein phosphatase, Mg2+/Mn2+ dependent 1J | | A0A4X1T6J6 | |
|  |  | Protein S100 | | A0A4X1UY31 | |
|  |  | Pyridoxal kinase | | A0A286ZIG4 | |
|  |  | Pyruvate kinase | | A0A0B8S031 | |
|  |  | RAB7A, member RAS oncogene family | | A0A287BQX8 | |
|  |  | Radixin | | A0A286ZJJ2 | |
|  |  | Ras GTPase-activating-like protein IQGAP1 | | A0A480LDL3 | |
|  |  | Ras-related protein Rab-14 | | A0A287BPB8 | |
|  |  | Septin | | A0A173G6G9 | |
|  |  | Serine/threonine kinase 24 | | A0A287B0C4 | |
|  |  | Small monomeric GTPase | | A0A4X1SFI5 | |
|  |  | Sodium/glucose cotransporter 1 | | A0A140H1A0 | |
|  |  | Sorbitol dehydrogenase | | A0A480UJV9 | |
|  |  | STEAP family member 4 | | A0A286K202 | |
|  |  | Thymosin beta | | A0A286ZVG3 | |
|  |  | Transket_pyr domain-containing protein | | A0A4X1VH46 | |
|  |  | Transmembrane channel-like protein | | A0A287A0L8 | |
|  |  | Triosephosphate isomerase | | A0A288CFT0 | |
|  |  | Tr-type G domain-containing protein | | A0A286ZUI3 | |
|  |  | Tubulin beta chain | | A0A480F1D4 | |
|  |  | Tyrosine 3-monooxygenase/tryptophan 5-monooxygenase activation protein, beta | | A0A0B8RVD2 | |
|  |  | Ubiquitin C | | A0A0B8RT13 | |
|  |  | Uncharacterized protein | | A0A287ALA0 | |
|  |  | Uncharacterized protein | | A0A4X1TV41 | |
|  |  | Uncharacterized protein | | A0A4X1VKL9 | |
|  | | 15S Mg(2+)-ATPase p97 subunit | | A0A286ZSB5 | |
| **C-sows and AI-sows** | 60S ribosomal protein L4 | | A0A0B8RT95 | |  |
|  | 60S ribosomal protein L9 | | A0A287AW71 | |  |
|  | Actin, gamma 1 | | A0A0B8RTA2 | |  |
|  | AHNAK nucleoprotein | | A0A287A608 | |  |
|  | Alkaline phosphatase | | A0A287BSC3 | |  |
|  | Annexin | | A0A286ZJV6 | |  |
|  | Atriopeptidase | | A0A287BPD6 | |  |
|  | BRO1 domain-containing protein | | A0A287A5B4 | |  |
|  | Cadherin-13 | | A0A287ALP4 | |  |
|  | D-3-phosphoglycerate dehydrogenase | | A0A480HK41 | |  |
|  | Deleted in malignant brain tumors 1 protein | | A0A480JBS1 | |  |
|  | Dynein cytoplasmic 1 heavy chain 1 | | A0A287B9W3 | |  |
|  | Ectonucleoside triphosphate diphosphohydrolase 1 | | A0A0B8RZL3 | |  |
|  | Glutathione hydrolase 1 proenzyme | | A0A480QXQ9 | |  |
|  | Glyceraldehyde-3-phosphate dehydrogenase | | A0A076KRX0 | |  |
|  | Interferon induced transmembrane protein 3 | | A0A0B8RSR9 | |  |
|  | Low-density lipoprotein receptor-related protein 2 | | A0A287AZ36 | |  |
|  | LPS-responsive vesicle trafficking, beach and anchor containing | | A0A0B8RTI4 | |  |
|  | Myosin-9 | | A0A480IS92 | |  |
|  | Rab GDP dissociation inhibitor | | A0A287B4B7 | |  |
|  | Rac family small GTPase 3 | | A0A286ZSH1 | |  |
|  | Ras homolog family member A | | A0A4X1ST20 | |  |
|  | Riboflavin kinase | | A0A4X1VWY3 | |  |
|  | Tubulin alpha chain | | A0A4X1W776 | |  |
|  | Ubiquitin C | | A0A0B8RT13 | |  |
|  |  | | A0A286ZT94 | |  |

**Supplementary table 4.** Exclusive and common proteins identified in extracellular vesicles from uterine fluid (uEVs) in sows (n=20) inseminated with different semen doses from boars (n = 6) according to the accumulative seminal fraction(s) used for artificial insemination (AI): F1, composed by the sperm-rich fraction (SRF); F2, composed by F1 plus the intermediate fraction; F3, composed by F2 plus the post-SRF.

| **Experimental group** | **Protein name** | **Accession number** |
| --- | --- | --- |
| **AI-F1** | 14_3_3 domain-containing protein | A0A4X1UM41 |
|  | 2',3'-cyclic-nucleotide 3'-phosphodiesterase | A0A286ZZ12 |
|  | 60S acidic ribosomal protein P2 | A0A287B7U0 |
|  | Actin, gamma 1 | A0A0B8RTA2 |
|  | Actin-depolymerizing factor | A0A286ZIC1 |
|  | Adseverin | A0A287A900 |
|  | Calcium-activated neutral proteinase 1 | A0A480EV23 |
|  | Calmodulin 3 | A0A287AHS0 |
|  | CCT-alpha | A0A287AJY2 |
|  | Cystathionine beta-synthase | A0A287AQV4 |
|  | Dynein cytoplasmic 1 heavy chain 1 | A0A287B9W3 |
|  | Elongation factor 2 | A0A287AWI9 |
|  | Epidermal growth factor receptor pathway substrate 8 | A0A287BJX5 |
|  | Ezrin | A0A480UUL9 |
|  | Glia maturation factor | A0A287A6Y3 |
|  | Glucose-6-phosphate isomerase | A0A480S836 |
|  | Glutaredoxin | F2XWZ9 |
|  | Guanine nucleotide binding protein (G protein), beta polypeptide 1 | A0A0B8RZJ8 |
|  | Heat shock protein HSP 90-alpha | A0A287AQK7 |
|  | Integrin-associated protein | A0A286ZQ17 |
|  | Interferon-induced transmembrane protein 1 | A0A142EGG5 |
|  | Mitogen-activated protein kinase | A0A286ZPF5 |
|  | Phosphoglycerate mutase | A0A287AJQ2 |
|  | Phosphoserine aminotransferase | A0A480S1F9 |
|  | Pyridoxal kinase | A0A286ZIG4 |
|  | Pyruvate kinase | A0A0B8S031 |
|  | RAB7A, member RAS oncogene family | A0A287BQX8 |
|  | Radixin | A0A286ZJJ2 |
|  | Ras homolog family member A | A0A4X1ST20 |
|  | Ras-related protein Rab-14 | A0A287BPB8 |
|  | Septin | A0A173G6G9 |
|  | Sorbitol dehydrogenase | A0A480UJV9 |
|  | Transket_pyr domain-containing protein | A0A4X1VH46 |
|  | Triosephosphate isomerase | A0A288CFT0 |
| **AI-F2** | 14-3-3 protein zeta/delta | A0A480PLY3 |
|  | DnaJ heat shock protein family (Hsp40) member A1 | A0A4X1UQS4 |
|  | Adenylyl cyclase-associated protein | A0A0B8S0B1 |
|  | EPS8 like 2 | A0A286ZVR9 |
|  | Heterogeneous nuclear ribonucleoprotein K | A0A4X1UTE2 |
|  | Na(+)-dependent phosphate cotransporter 2B | A0A4X1V982 |
| **AI-F3** | Ammonium transporter Rh type C | A0A4X1U0V5 |
|  | ATP-binding cassette sub-family G member 2 | A0A4X1U7U2 |
|  | Filamin B | A0A286ZPG4 |
|  | Glutathione hydrolase 1 proenzyme | A0A480QXQ9 |
|  | Pendrin | A0A480E679 |
|  | Proline rich transmembrane protein 1B | A0A287ADX3 |
|  | STEAP family member 4 | A0A286K202 |
| **AI-F1, AI-F2, and AI-F3** | 15S Mg(2+)-ATPase p97 subunit | A0A286ZSB5 |
|  | 2-phospho-D-glycerate hydro-lyase | A0A0B8RSY9 |
|  | 3-hydroxyacyl-[acyl-carrier-protein] dehydratase | A0A480M011 |
|  | 60S ribosomal protein L4 | A0A0B8RT95 |
|  | Alkaline phosphatase | A0A287BSC3 |
|  | Annexin | A0A286ZJV6 |
|  | Atriopeptidase | A0A287BPD6 |
|  | Brain-specific angiogenesis inhibitor 1-associated protein 2 | A0A287BKX8 |
|  | BRO1 domain-containing protein | A0A287A5B4 |
|  | Cadherin-13 | A0A287ALP4 |
|  | cAMP-dependent protein kinase type II-alpha regulatory subunit | A0A4X1T0V7 |
|  | Chloride intracellular channel protein | A0A0B8S0A2 |
|  | Cofilin, non-muscle isoform | A0A286ZVE7 |
|  | D-3-phosphoglycerate dehydrogenase | A0A480HK41 |
|  | Ectonucleoside triphosphate diphosphohydrolase 1 | A0A0B8RZL3 |
|  | Glyceraldehyde-3-phosphate dehydrogenase | A0A076KRX0 |
|  | HATPase_c domain-containing protein | A0A286ZKC5 |
|  | LPS-responsive vesicle trafficking, beach and anchor containing | A0A0B8RTI4 |
|  | MARCKS like 1 | A0A287B4N8 |
|  | Mucin 4, cell surface associated | A0A287B5M2 |
|  | Myristoylated alanine rich protein kinase C substrate | A0A287BRL8 |
|  | Na(+)/H(+) exchange regulatory cofactor NHE-RF | A0A287AAH4 |
|  | Neuroblast differentiation-associated protein AHNAK isoform X1 | A0A480Z467 |
|  | Niban-like protein 1 | A0A0B8RSY4 |
|  | PDZ domain containing 1 | A0A287BAJ3 |
|  | PKS_ER domain-containing protein | A0A4X1TWX8 |
|  | Protein GNAS isoform GNASS | A0A480UPA5 |
|  | Rab GDP dissociation inhibitor | A0A287B4B7 |
|  | Rac family small GTPase 3 | A0A286ZSH1 |
|  | Riboflavin kinase | A0A4X1VWY3 |
|  | Sodium/glucose cotransporter 1 | A0A140H1A0 |
|  | Transmembrane channel-like protein | A0A287A0L8 |
|  | Tubulin alpha chain | A0A4X1W776 |
|  | Tubulin beta chain | A0A480F1D4 |
|  | Uncharacterized protein | A0A286ZT94 |
|  | Uncharacterized protein | A0A287ALA0 |
|  | Uncharacterized protein | A0A4X1TV41 |
|  | Uncharacterized protein | A0A4X1VKL9 |
| **AI-F1 and AI-F2** | 60S ribosomal protein L9 | A0A287AW71 |
|  | AHNAK nucleoprotein | A0A287A608 |
|  | Charged multivesicular body protein 6 | A0A4X1UDW0 |
|  | Cytosolic non-specific dipeptidase isoform X1 | A0A480SKP2 |
|  | Deleted in malignant brain tumors 1 protein | A0A480JBS1 |
|  | G protein subunit alpha i3 | A0A4X1TDW5 |
|  | Interferon induced transmembrane protein 3 | A0A0B8RSR9 |
|  | Maillard deglycase | A0A4X1W8H4 |
|  | Non-specific serine/threonine protein kinase | A0A287APG0 |
|  | Peroxiredoxin-1 | A0A286ZND5 |
|  | Plastin 1 | A0A287APD4 |
|  | Protein phosphatase, Mg2+/Mn2+ dependent 1J | A0A4X1T6J6 |
|  | Protein S100 | A0A4X1UY31 |
|  | Serine/threonine kinase 24 | A0A287B0C4 |
|  | Small monomeric GTPase | A0A4X1SFI5 |
|  | Thymosin beta | A0A286ZVG3 |
|  | Tr-type G domain-containing protein | A0A286ZUI3 |
|  | Tyrosine 3-monooxygenase/tryptophan 5-monooxygenase activation protein, beta | A0A0B8RVD2 |
|  | Ubiquitin C | A0A0B8RT13 |
| **AI-F1 and AI-F3** | Actin-like protein 3 | A0A0B8RZS4 |
|  | Myosin-10 | A0A480HB69 |
| **AI-F2 and AI-F3** | 40S ribosomal protein S8 | A0A4X1SUR2 |
|  | DNAJ heat shock protein family (Hsp40) member A2 | A0A4X1TXQ3 |
|  | Heat shock protein family A (Hsp70) member 8 | A0A286ZPC8 |
|  | Low-density lipoprotein receptor-related protein 2 | A0A287AZ36 |
|  | Myosin-9 | A0A480IS92 |
|  | Ras GTPase-activating-like protein IQGAP1 | A0A480LDL3 |
|  | Ras homolog family member A | A0A4X1ST20 |
